# Kinetic Locking of pH-Sensitive Complexes for Mechanically Responsive Polymer Networks

**DOI:** 10.1021/jacs.5c09897

**Published:** 2025-09-08

**Authors:** Stephen J.K. O’Neill, Yuen Cheong Tse, Zehuan Huang, Xiaoyi Chen, Jade A. McCune, Oren A. Scherman

**Affiliations:** Melville Laboratory for Polymer Synthesis, Yusuf Hamied Department of Chemistry, 2152 University of Cambridge, Lensfield Road, Cambridge CB2 1EW, U.K.

## Abstract

Achieving sensitive and reversible responsivity over physiologically relevant pH ranges (4.5–7.5) remains of great interest for the design of next-generation autonomous drug delivery devices. Developing molecular interactions that are responsive within this pH range would enable targeted drug delivery at tumor sites or within inflamed or arthritic joints, where these changes in pH occur. Here, we demonstrate pH-responsive molecular interactions by the kinetic locking of host–guest complexes. Employing these complexes as dynamic crosslinks within polymer networks gives rise to materials with highly pH-responsive mechanical and viscoelastic properties. These systems further exhibit pH-dependent release of cargo, offering a self-responsive approach toward targeted drug delivery.

## Graphical abstract

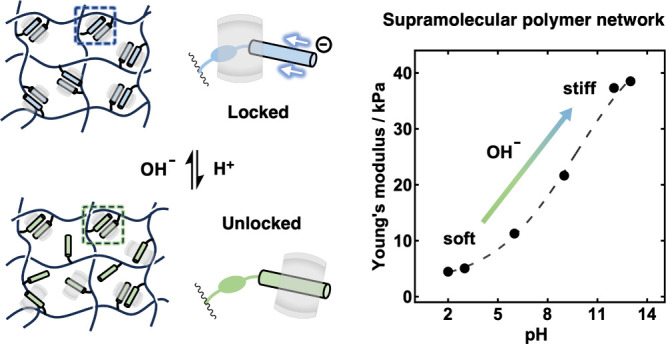

The ability to kinetically lock molecules in response to external stimuli has wide utility both in natural and synthetic systems.^1,2^ In biological systems, signal transduction pathways rely on the calcium/calmodulin-dependent protein kinase II (CaMKII) to switch between locking states, whereby Ca^2+^-loaded calmodulin wraps around peptide segments and triggers dissociation from active ATP sites.^3,4^ Lockable synthetic systems have since been developed exhibiting rapid and reversible locking of molecules following heat,^5^ light,^6^ or chemical,^7^ stimuli. Of particular interest for biomedical applications are pH-responsive systems, toward targeted drug-release at cancerous tumors or arthritic joints.^8,9^ pH-responsive drug release has thus far relied on self-assembled nanoparticles or pH-sensitive chemical bonds.^10–12^ Such strategies, however, suffer from a lack of control over disassembly rate, limited reversibility, and a narrow range of responsive pH.^13,14^

Cucurbit­[*n*]­uril (CB­[*n*]) host–guest interactions have been widely employed for supramolecular oligomers,^15^ polymers,^16^ hydrogels,^17^ functional interfaces,^18^ and molecular separators.^19,20^ A broad range of accessible binding affinities (10^2^–10^15^ M^–1^) can be achieved on account of hydrophobic and ion-dipole interactions with guest molecules.^21–23^ Many of these host–guest interactions can be designed to respond to external stimuli, whereby changes in the chemical structures of the guest molecules directly affect binding strength through the application of light,^24,25^ pH,^26–30^ or redox.^31,32^ In the body, pH changes are tightly regulated (pH 4.5–7.5) and often serve as indicators of physiological changes such as inflammation, where localized acidosis can signal tissue stress or immune system activation. We envisaged that kinetic-locking of host–guest complexes could be achieved in accordance within this narrow pH range, offering the potential to realize rapid, reversible, and sensitive stimuli-responsive materials.

Here, we develop pH-responsive molecular interactions through kinetic locking of CB­[*n*] (*n* = 7, 8) host–guest complexes (Figure 1). Through careful design of the guest moiety BPI/BPI^–^, the resulting complex (BPI^–^·CB­[7]) demonstrates pH-dependent kinetic locking based on ion-dipole repulsive forces, trapping the host macrocycle in place, thus inhibiting dethreading.

**Figure 1:**
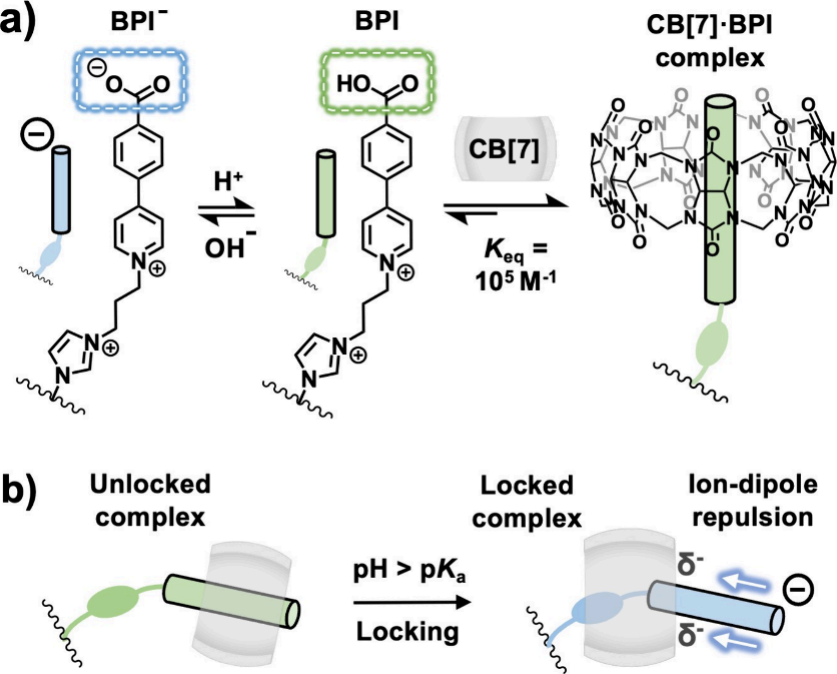
(a) pH-responsive BPI guest and supramolecular complex formation with a CB[7] host. (b) pH-dependent locking of the host–guest complex. Counterions omitted for clarity.

The guest moiety BPI, bearing a terminal carboxylic acid group and two cationic moieties (imidazolium and pyridinium) was designed and synthesized (Figure 1a). Upon an increase in pH, the carboxylic acid deprotonates, affording the carboxylate (BPI^–^). Formation of the carboxylate gives rise to ion-dipole repulsion with the δ-negative rim of the CB­[*n*] (*n* = 7, 8) carbonyl portal, hindering dissociation and kinetically locking the guest in place (see Figure 1b and Figure S20). Meanwhile, the phenyl pyrdinium and imidazolium ensure high binding affinity on account of hydrophobic interactions and ion-dipole attractive forces.^34,35^ The high binding strength of the BPI moiety (*K*_eq_ > 10^5^ M^–1^) with CB[7] was confirmed through isothermal titration calorimetry (ITC) at pH 3, binding in a 1:1 ratio (Figure S26).

The locking mechanism was demonstrated through competitive binding experiments as shown in Figure 2a. The guest molecule **TEG-BPI** was introduced to sterically restrict dethreading of CB[7] at the opposite end from the carboxylic acid through introduction of the bulky tris­(tri­(ethylene glycol monomethyl ether)) benzene stopper motif (Figure S1). In the protonated state (pH 3), the host molecule CB[7] can dethread over the carboxylic acid, and thereby be available for complexation with alternative competitive guest molecules. Meanwhile, if the pH is above the p*K*_a_ of the complex, the carboxylate locks the CB[7] in place as a pseudorotaxane, and the host becomes inaccessible for further binding.

**Figure 2:**
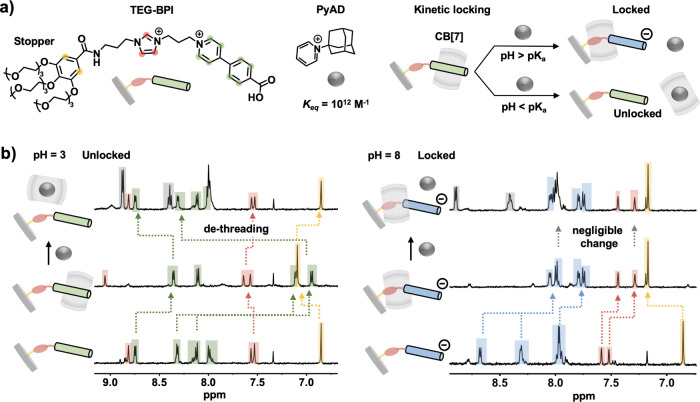
(a) Experimental investigation with end-capped BPI into locking mechanism. (b) ^1^H NMR spectra (D_2_O, 298 K) of the complex in the unlocked state (left), and the locked state (right) before and after introduction of a PyAD competitive guest.

Through ^1^H NMR spectroscopy, clear dethreading of CB[7] from the TEG-BPI is observed following the addition of excess pyridinium adamantane (PyAD) competitive guest to the complex in the unlocked state (pH = 3), Figure 2b. The peaks corresponding to the arylpyridinium group on TEG-BPI shift downfield as they are no longer shielded within the CB[7] cavity, returning to the shift values observed for free TEG-BPI. Conversely at pH = 8, minimal change in the ^1^H NMR is observed following the addition of PyAD consistent with the TEG-BPI^–^·CB­[7] remaining in the locked state. Given the ultra high binding affinity of PyAD for CB[7] (2 × 10^12^ M^–1^),^36^ lack of dissociation between the CB[7] host and TEG-BPI^–^ guest confirms the exceptional locking ability of the BPI system. Further, no dissociation was observed either over extended time periods (Figure S23), as well as at elevated temperatures (Figure S28).

Employing the larger host molecule CB[8] enables simultaneous complexation of two BPI guest derivatives within the macrocycle, Figure 3a. We postulated that integration of the lockable complex as cross-links within a polymer network would result in pH-responsive materials, Figure 3c. To realize such materials, the guest monomer **VBPI** was synthesized, whereby the addition of a vinyl group to the BPI guest enables facile photoinduced free radical polymerization (FRP) with an acrylamide monomer (Figure S30).

**Figure 3:**
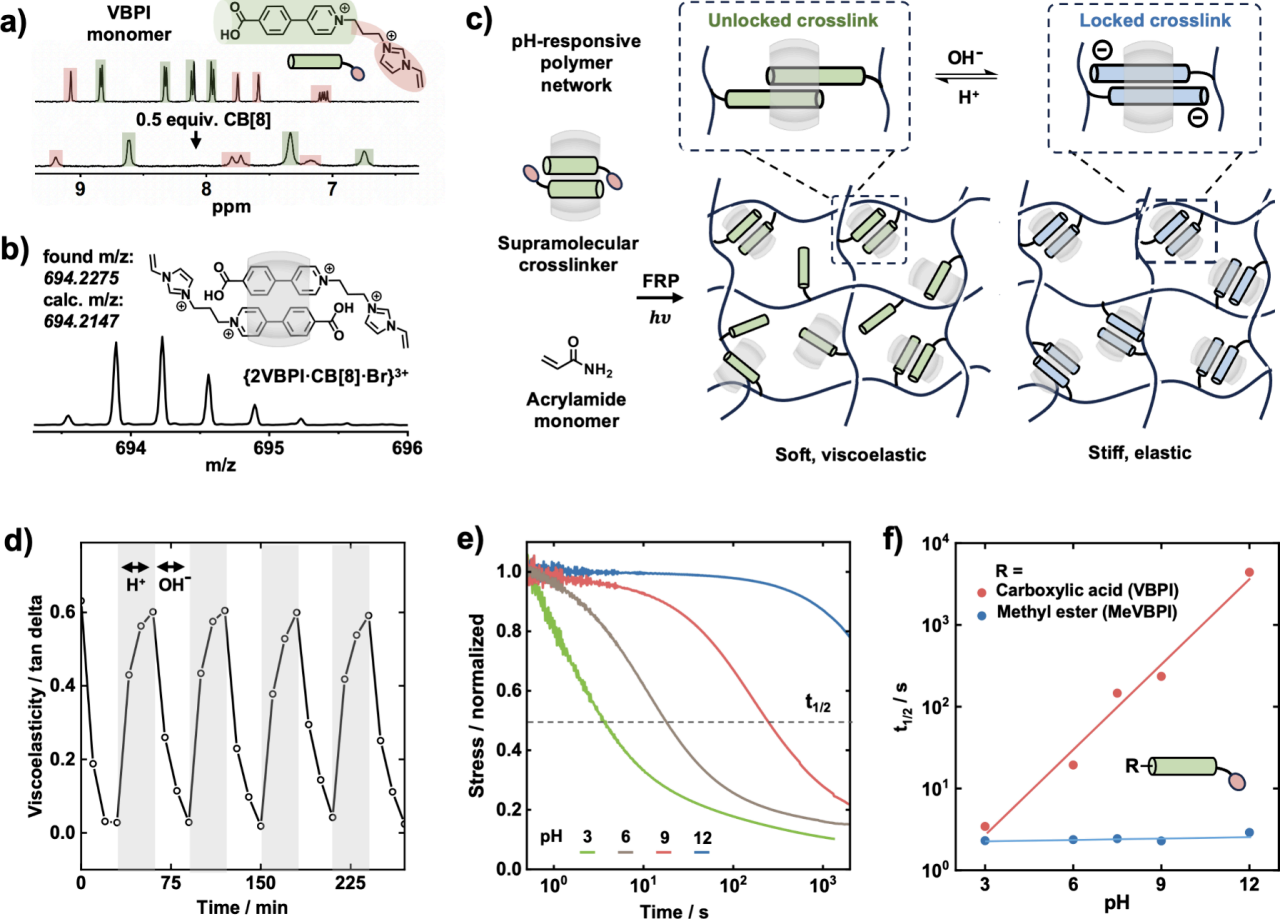
(a) ^1^H NMR spectra (D_2_O, 298 K) of the VBPI guest (top), and after 2:1 complexation with CB[8] (bottom). (b) HR ESI-MS spectrum (from H_2_O) of the 2VBPI·CB[8] complex. (c) Preparation and switching pH-responsive polymer network. (d) Viscoelasticity (tan δ) measurements of the polymer network cycling between acidic and basic pH. (e) Rheological stress relaxation at varying pH values. (f) Stress relaxation *t*_1/2_ for polymer networks with VBPI and MeVBPI complexes as crosslinks.

The formation of a 2:1 2VBPI·CB[8] complex was confirmed by ^1^H NMR spectroscopy (Figure 3a). Upon addition of 0.5 equiv of CB[8] to VBPI, the peaks corresponding to the phenylpyridinium protons of VBPI (green) showed significant upfield shifts, indicating increased shielding as a result of encapsulation within the CB[8] cavity. Importantly, no upfield shifts were observed for the vinyl protons (red), indicating that they are not encapsulated within the CB[8] cavity and remain accessible for polymerization.

High-resolution electrospray ionization mass spectrometry (ESI-MS) was also carried out to further probe formation of the 2VBPI·CB[8] complex (Figure 3b). An intense ion peak was observed at *m*/*z* = 694.2275 ({2VBPI·CB[8]·Br}^3+^) with a Δ*m*/*z* of 0.3335, highlighting the stability of the 2:1 complex. A high overall binding affinity (*K*_1_ *K*_2_ > 10^10^ M^–2^) for VBPI complexation with CB[8] was determined through ITC measurements (Figure S27), indicating suitability for robust supramolecular cross-linked materials formation.^37,38^ Interestingly, an increase of 0.52 p*K*_a_ units was observed for the CB[8] complex over the VBPI monomer itself (Figure S29).^39,40^

The resultant VBPI polymer networks showed highly pH-responsive viscoelastic properties and completely reversible switching between acidic and basic environments (Figure 3d). Following immersion in an acidic solution (pH 3), the materials exhibit highly viscoelastic properties with a tan δ (ratio of loss to storage modulus) > 0.6, typical for a supramolecular hydrogel with dynamic crosslinks.^41,42^ As the environmental pH is increased to a value of 12, the tan δ rapidly drops (∼0.02 after 30 min), indicating a change from dynamic to static polymer networks on account of the kinetic locking of the CB[8] cross-links. The materials demonstrated exceptional reversibility over ten cycles, between static (pH 12) and dynamic (pH 3) states.

Stress relaxation and frequency-sweep measurements were employed to investigate pH-dependent cross-link kinetics in the polymer networks (see Figure 3d and Figure S31). Under acidic conditions (pH 3), the VBPI polymer network displays rapid stress relaxation within 10 s (Figure 3e). Fast stress relaxation can be attributed to the dissociation kinetics of the VBPI crosslinks in their unlocked state, and is typical for CB[8]-based supramolecular polymer networks.^43,44^ Conversely, under basic conditions (pH 12), minimal stress relaxation is observed for the VBPI polymer networks across 1000 s, typical for highly cross-linked or covalent networks.^45^ The polymer networks further exhibit both a temperature- and frequency-independent viscoelastic response across multiple time scales under basic conditions, indicating kinetic locking of the crosslinks with arrested dissociation, Figure S34.

To ensure that the pH-responsive behavior derives from kinetic locking of the supramolecular crosslinks, control experiments were performed using a methyl ester derivative of VBPI (MeVBPI) for the formation of a CB[8] cross-linked polymer network (see Figure 3f and Figure S33). As opposed to carboxylic acid, methyl ester does not readily undergo protonation or deprotonation within physiologically relevant pH ranges,^46^ and therefore no locking mechanism is expected to occur. The stress relaxation half-time (*t*_1/2_, defined as the time taken for the stress to drop below half of the initial value), was compared for both networks across a range of pH values. While the VBPI polymer network exhibited highly pH-dependent stress relaxation behavior spanning over 3 orders of magnitude (*t*_1/2_ ≈ 2–4000 s), the MeVBPI network displayed no pH-dependent behavior. Rather, it maintained a stress relaxation response consistent with that of viscoelastic supramolecular materials (*t*_1/2_ ≈ 2 s), confirming that the carboxylic acid moiety is indeed responsible for the pH responsivity.

The VBPI polymer network further displays highly pH-dependent mechanical properties (Figures 4a–c). Through tensile deformation experiments, it was observed that a significant increase in force was required to deform the materials at higher pH (Figure 4a). The difference in tensile strength can be quantified by the Young’s modulus (Figure 4b), which increases by an order of magnitude from 4 kPa (pH 3) to 40 kPa (pH 12). The elongation at break of the materials substantially diminishes with increasing pH (see Figure 4b and Figure S38), consistent with a transition from a dynamic to static polymer network.^47^

**Figure 4:**
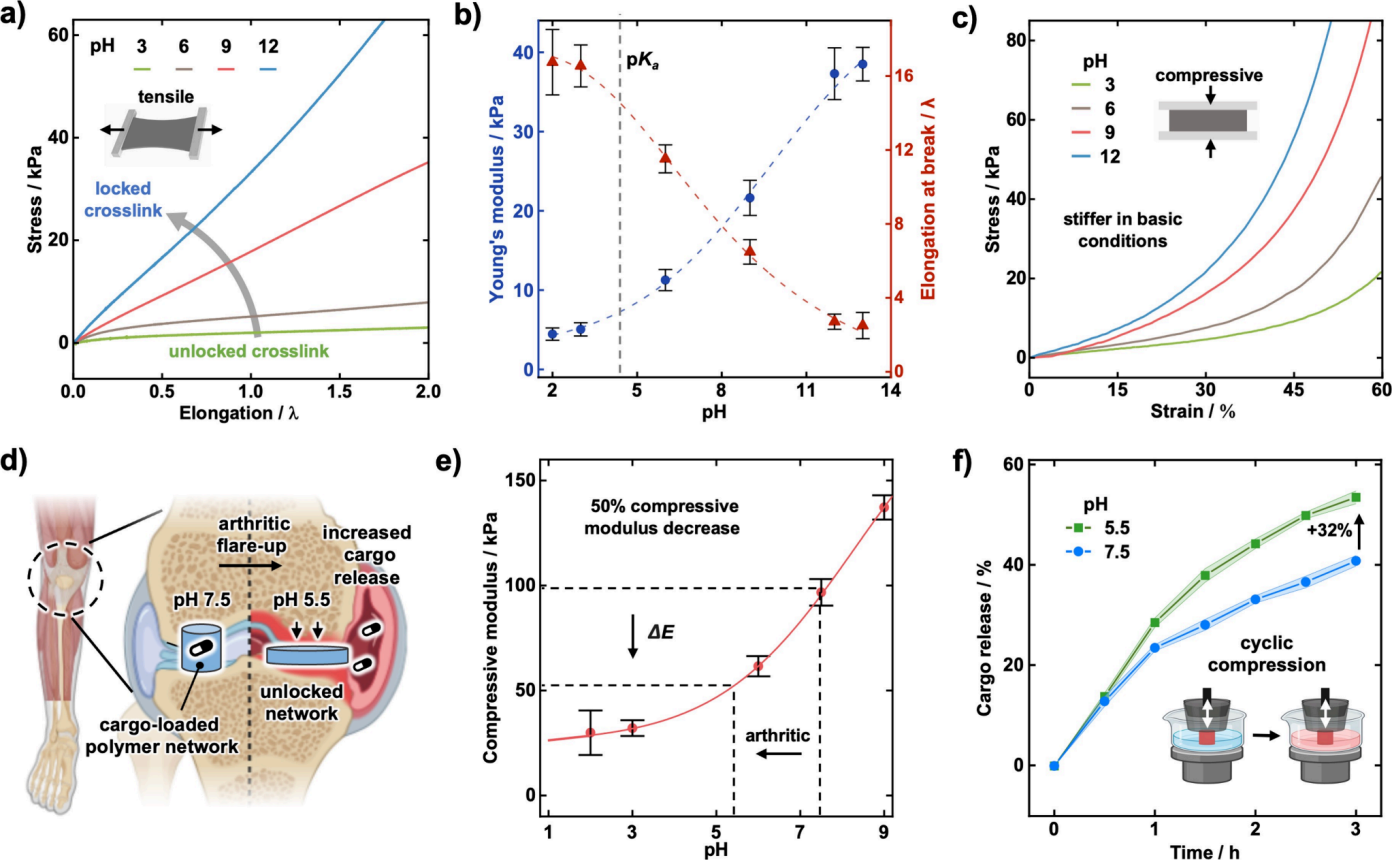
(a) Stress–strain tensile curves of the polymer networks. (b) Young’s modulus and stretchability of the polymer networks across a pH range. (c) Stress–strain compressive curves of the polymer networks. (d) Schematic of a knee joint with transient cargo delivery during an arthritic flareup.^33^ (e) Compressive modulus of the polymer network across a pH range. (f) Cargo release during cyclic compression at pH 5.5 and pH 7.5.

The compressibility of the materials follow the same trend, requiring more force for deformation at higher pH (Figure 4c). Compressing the materials to 60% required over seven times more force at pH 12 (162 kPa) compared to pH 3 (21 kPa). As the pH increases, the cross-links transition to a locked state, arresting their dissociation and requiring more force for polymer deformation. The mechanical properties showed complete reversibility switching between unlocked and locked states (Figure S39).

The suitability of these materials toward transient, site-specific drug delivery for arthritis was evaulated (Figures 4d–f). Rheumatoid and osteoarthritis are associated with significant drops in endogenous pH during inflammatory flareups, with a pH value as low as 5.5 reported for the cartilage surface (Figure 4d).^48^ We envisioned that loading VBPI polymer networks with cargo, such as anti-inflammatory or immunosuppressive drugs, would enable an autonomous drug-delivery system responsive to arthritic flareups (Figure 4d). By implanting the drug-loaded polymer networks within the joint as artificial cartilage, decreases in pH would give rise to increased deformation on account of the highly responsive compressive strength of the materials (Figure 4e). Unlike systems that require external stimuli (e.g., temperature, light), this material offers a self-regulated release platform that responds to endogenous pH.

As a proof of concept, the polymer networks were loaded with cargo in the form of a small molecule dye (Sulforhodamine B), and release from the polymer network was measured overtime during compressive cycling at different environmental pH values (see Figure 4f and Figure S40). It was found that, at the pH of an arthritic joint (pH 5.5), a 32% increase in cargo release was observed after 3 h, compared to that of a healthy joint (pH 7.5). Such findings are highly promising toward autonomous site-specific cargo delivery, improving drug potency and minimizing side effects.

In conclusion, we have demonstrated unprecedented kinetic locking behavior of CB­[*n*] host–guest complexes. Through careful design of a carboxylic acid containing guest molecule, modulation of pH-dependent ion-dipole interactions result in switchable locking with the CB­[*n*] host. Integrating such supramolecular complexes into a polymer network gives rise to significant and reversible viscoelastic and mechanical responses across physiologically relevant pH ranges. The ability to drastically vary material properties within physiologically relevant pH ranges offers new avenues toward future biomedical applications, in particular smart, autonomous drug delivery for sustained and controlled release of anti-inflammatory or immunosuppressive drugs for treating diseases such as rheumatoid arthritis.

## Supplementary Material



## Notes

Materials and instrumentation, synthesis and characterization of molecules, binding studies, determination of p*K*_a_, polymer network formation, mechanical and rheological characterization, pH-dependent cargo release study (PDF)
