# Supplementary material for: Kinetic Locking of pH-Sensitive Complexes for Mechanically Responsive Polymer Networks
Source: J Am Chem Soc. 2025 Sep 8;147(37):33337–42. doi: 10.1021/jacs.5c09897 (PMC12447493; doi:10.1021/jacs.5c09897)
Supplement: Supplementary file 1 [file ja5c09897_si_001.pdf]

Supporting information
Kinetic locking of pH-sensitive complexes for mechanically responsive polymer networks

Stephen J.K. O'Neill, Yuen Cheong Tse, Zehuan Huang, Xiaoyi Chen, Jade A. McCune, and Oren A. Scherman*

Melville Laboratory for Polymer Synthesis, Yusuf Hamied Department of Chemistry, University of Cambridge, Lensfield Road, Cambridge CB2 1EW, UK.

E-mail: oas23@cam.ac.uk

Contents

1 Materials & Instrumentation **S2**

2 Synthesis & Characterisation of Molecules **S5**

3 Spectral Characterisation of Previously Unreported Molecules **S14**

4 Binding Studies **S22**

5 Variable temperature investigation **S31**

6 Determination of pK_a **S33**

7 Polymer Network Formation **S34**

8 Mechanical & Rheological Characterisation **S36**

9 pH-dependent Cargo Release Study **S45**

References **S46**

1 Materials & Instrumentation

1 .1 Materials

Unless otherwise stated, all the chemicals used in the present work were purchased from Sigma Aldrich and used directly without further purification: acrylamide (for molecular biology, 99%, high-performance liquid chromatography grade (HPLC)), 1-vinylimidazole (99%), 4-(pyridin-4-yl)benzoic acid, 1,3-Dibromopropane (98%), acetonitrile (HPLC, 99.9%), 4-(Bromomethyl)phenylacetic acid (99%), 4-(Dimethylamino)-pyridine (DMAP, 99%), 1-Ethyl-3-(3-dimethylaminopropyl)carbodiimide (EDC, 99%), methyl 4-(pyridin-4-yl)benzoate (99%), diethyl ether (ACS reagent, 99%), N-hydroxy-succinimide (NHS, 99%), deuterium oxide (D_2O, D 99.8%), nitrogen, 2-hydroxy-4-(2-hydroxyethoxy)-2-methylpropiophenone (photoinitiator, I-2959, 98%). Cucurbit[8]uril (CB[8]) was prepared and purified using a previously reported method.[S1,S2] Milli-Q water was obtained from a Milli-Q Integral Water Purification System (18.2 MΩ·cm). Unless otherwise noted, all the sample solutions were prepared in D_2O or Milli-Q H_2O under heating and ultrasonication.

1 .2 Nuclear Magnetic Resonance (NMR) Spectroscopy

^{1}H NMR spectra were acquired in D_2O at 298.15 K on a Bruker AVANCE 500 or 700 (500 MHz, 700 MHz) apparatus as specified. Chemical shifts for proton peaks in ^{1}H NMR were referenced to the residual solvent peak (HDO) at 4.79 ppm. For variable temperature NMR, the residual solvent peak was referenced according to a previously reported temperature relationship.[S3] ^{13}C NMR spectra were acquired in D_2O at 298.15 K on a Bruker AVANCE 700 apparatus with TCI Cryoprobe system (700 MHz). All the sample solutions for NMR experiments were prepared in pure D_2O at 0.2 mM - 1.0 mM for ^{1}H & and 10.0 mM for ^{13}C NMR.

1 .3 Electrospray Ionization Mass Spectrometry (ESI-MS)

ESI-MS spectra were acquired on a Thermo Fisher Q Exactive Orbitrap mass spectrometer with a nanospraying ionization source, using borosilicate emitters as the one-off needles to inject the sample solutions into the MS apparatus. ESI-MS experiments were performed in the positive ion mode at the m/z range from 150–2000 under the working temperature at 320 °C and the capillary voltage of 1.5 kV. The m/z value was calibrated in advance by a standard test sample before all the MS characterization. All

the sample solutions used in MS experiments were prepared in Milli-Q water at 1.0 mM, and all the obtained data were analyzed in Origin 10.0 software.

1 .4 Isothermal Titration Calorimetry (ITC)

ITC experiments were conducted on a Malvern MicroCal Auto-ITC200 apparatus at 298.15 K in Milli-Q H_2O. In a typical titration, the host molecule (CB[8]) was loaded in the sample cell at a concentration of 0.05 mM, and the guest monomer (BPyVI) was loaded in the syringe at a 20-fold higher concentration of 1.0 mM. One titration experiment consisted of 1 injection of 0.6 mL and 32 consecutive injections of 1.2 mL with 90 s intervals between injections. The first data point was removed before data analysis as it may contain contamination. The resultant ITC curves were fitted by the sequential binding model, using Malvern MicroCal Analysis Centre software to gain thermodynamic information. All the titrations were repeated three times to provide the mean values of thermodynamic and kinetic parameters with their corresponding error bars (s.d. n=3).

1 .5 Rheology

Rheological characterisation was carried out by a Discovery Hybrid Rheometer (DHR)-2, TA Instruments, with a Peltier Plate for temperature control. The polymer network specimens were immersed in solutions of 1 x phosphate buffered saline (PBS) at each given pH for 6 hours (where the pH of the solution was controlled using NaOH or HCl), following which the samples were tested. All the measurements were conducted using a 20 mm parallel stainless steel plate geometry, and the necessary calibration for geometry was carried out before testing. Oscillatory frequency-sweep measurements were conducted at 1% strain in the frequency range from 0.1 to 100 rad s^{-1}. Stress-relaxation data was collected following a single step-strain of 10%, following which the stress was measured over time. Rheology was performed during cycling between acidic and basic pH by measuring tan δ at different time points at a frequency of 0.1 rad s^{-1}. The data was collected at 293.15 K and analyzed by TRIOS software, TA Instruments.

1 .6 Mechanical Testing

Tensile tests were performed after SPN samples were immersed in solutions of 1 x phosphate buffered saline (PBS) at variable pH for 6 hours, where the pH was adjusted using NaOH or HCl. The samples were tested on a Instron machine (34TM-10) equipped

with a 50-N load cell at room temperature. A typical tensile test was performed by stretching a dumbbell-shaped specimen (following ISO4661-1 standard) at 10 mm min^{-1} until its breakage, to obtain stress-strain curves. Young's modulus and toughness are calculated from the slope and the area under the curve, respectively. Compressive tests were conducted on a Instron machine (34TM-10) equipped with 10-kN load cell at room temperature. In a typical compressive test, a cylinder specimen (20 mm diameter x 4 mm height) was placed between two stainless steel plates and compressed at a specific deformation rate (10 mm min^{-1}) to obtain stress-strain curves. Compression-retraction cycles were obtained by compressing cylinder specimens until the set strain of 60% and returning to the initial point.

2 Synthesis & Characterisation of Molecules

2 .1 TEG-BPI Synthesis

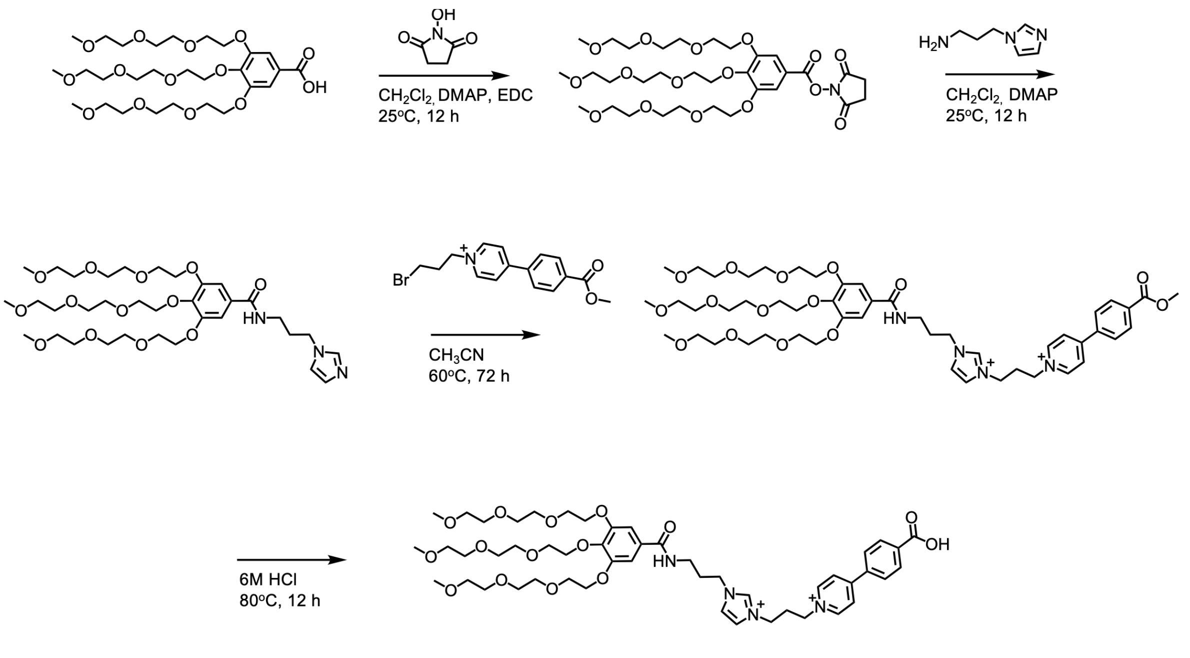

Figure S1 : Synthetic route for the preparation of TEG-BPI. Counterions have been omitted for clarity.

2 .1.1 Stopper NHS ester, TEG-NHS

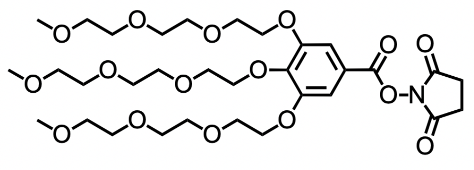

Figure S2 : Molecular structure of TEG-NHS.

3,4,5-tris[2-[2-(2-Methoxyethoxy)ethoxy]ethoxy]benzoic acid (ref: Chem. Commun. 2016, 52, 9009[S4]) (4.2 g, 6.90 mmol, 1.0 equiv) and N-hydroxysuccinimide (NHS) (1.19 g, 10.4 mmol, 1.5 equiv) were dissolved in anhydrous CH_2Cl_2 (30 mL) and cooled to 0 °C. Subsequently EDC·HCl (1.98 g, 10.4 mmol, 1.5 equiv) and DMAP (1.69 g, 13.8 mmol, 2.0 equiv) were added and the reaction mixture was stirred at room temperature overnight under N_2. The crude mixture in CH_2Cl_2 was washed with 1 M HCl (50 mL), saturated $NaHCO_3$ (50 mL) and H_2O (50 mL). The organic layer was dried with anhydrous $MgSO_4$, filtered and dried in vacuo to afford the product as a yellow oil (4.77 g, 98%).

^{1}H NMR (700 MHz, $CDCl_3$) δ 7.37 (s, 2H), 4.29–4.24 (m, 2H), 4.21–4.17 (m, 4H), 3.87–3.83 (m, 4H), 3.81–3.77 (m, 2H), 3.73–3.68 (m, 6H), 3.67–3.63 (m, 6H), 3.65–3.60 (m, 6H), 3.53 (ddd, J = 5.7, 3.3, 1.1 Hz, 6H), 3.36 (d, J = 1.3 Hz, 9H), 2.89 (s, 4H).

^{13}C NMR (176 MHz, $CDCl_3$) δ 169.22, 161.43, 152.62, 144.51, 119.39, 110.13, 72.60, 71.95, 71.94, 71.93, 70.87, 70.82, 70.71, 70.68, 70.59, 70.57, 70.55, 70.52, 69.57, 69.07, 59.02, 25.68 (some signals overlapping).

HRMS for {TEG-NHS}: found m/z = 706.3278, calculated m/z = 706.3286.

2 .1.2 Stopper imidazole, TEG-I

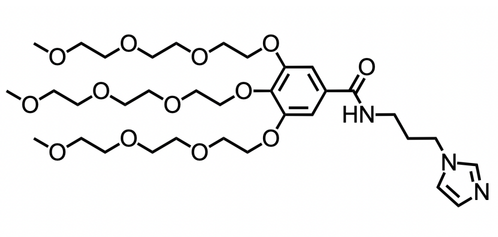

Figure S3 : Molecular structure of TEG-I.

1-(3-Aminopropyl)imidazole (169 uL, 1.42 mmol, 1.0 equiv) and DMAP (0.74 mL, 4.25 mmol, 3.0 equiv) were added to anhydrous CH_2Cl_2 (8 mL). A solution of TEG-NHS (1.0 g, 1.42 mmol, 1.0 equiv) in anhydrous CH_2Cl_2 (2 mL) was added and the reaction mixture was stirred at room temperature overnight under N_2. Subsequent purification with preparative TLC (10% CH_3OH in CH_2Cl_2) gave the product as colourless oil (534 mg, 53%).

^{1}**H NMR** (700 MHz, $CDCl_3$) δ 7.97 (s, 1H), 7.18 (s, 2H), 7.09 (t, J = 1.3 Hz, 1H), 7.05 (t, J = 1.4 Hz, 1H), 4.25–4.20 (m, 4H), 4.22–4.17 (m, 2H), 4.11 (t, J = 6.9 Hz, 2H), 3.84–3.81 (m, 4H), 3.78–3.75 (m, 2H), 3.71–3.69 (m, 6H), 3.65–3.61 (m, 14H), 3.56–3.50 (m, 6H), 3.35 (d, J = 15.5 Hz, 10H), 2.13 (p, J = 6.7 Hz, 2H).

^{13}**C NMR (176 MHz, $CDCl_3$)** δ 173.05, 167.38, 152.34, 141.37, 136.75, 129.32, 126.86, 119.43, 107.59, 72.30, 71.94, 71.88, 71.87, 70.60, 70.56, 70.55, 70.48, 70.43, 70.35, 69.73, 69.10, 65.86, 59.03, 58.97, 45.45, 37.07, 30.96, 25.47, 15.28 (some signals overlapping).

HRMS for {TEG-I}: found m/z = 716.3961, calculated m/z = 716.3964.

2 .1.3 1-(3-bromopropyl)-4-(4-(methoxycarbonyl)phenyl)pyridinium bromide, BPMe

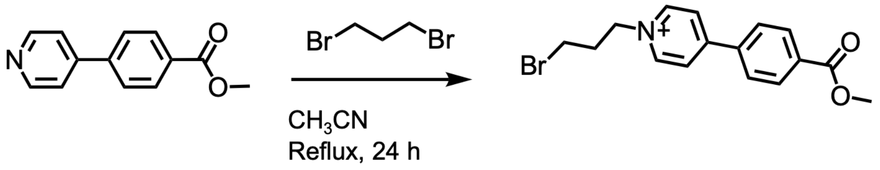

Figure S4 : Synthetic route for the preparation of BPMe. Counterions have been omitted for clarity.

methyl 4-(pyridin-4-yl)benzoate (0.64 g, 3 mmol, 1.0 equiv) and 1,3-dibromopropane (30.3 g, 150 mmol, 50 equiv) were added to CH_3CN (10 ml) and refluxed for 72 h. The resulting mixture was transferred to two centrifuge tubes, and ether was added (30 ml each) to afford the precipitate 1-(3-bromopropyl)-4-(4-(methoxycarbonyl)phenyl)-pyridinium bromide. The intermediate was collected as the precipitate by centrifugation at 10,000 rpm for 10 minutes, washed with ether (re-suspended in 40 ml ether, and centrifuged again to collect the precipitate), and dried *in vacuo* for use in the next step as a white solid (0.89 g, 72 %).

^{1}H NMR (700 MHz, D_2O) δ 8.97–8.92 (m, 2H), 8.39–8.35 (m, 2H), 8.20–8.16 (m, 2H), 8.03–7.98 (m, 2H), 4.83 (t, J = 7.1 Hz, 2H), 3.97 (s, 3H), 3.55 (t, J = 6.2 Hz, 2H), 2.64 (ddd, J = 13.3, 7.0, 6.0 Hz, 2H).

^{13}C NMR (176 MHz, D_2O) δ 168.33, 155.63, 144.61, 138.25, 132.27, 130.40, 128.25, 125.77, 59.33, 52.92, 32.66, 28.81.

HRMS for {MeVBPI}: found m/z = 334.0435, calculated m/z = 334.0437.

2 .1.4 TEG-BPIMe

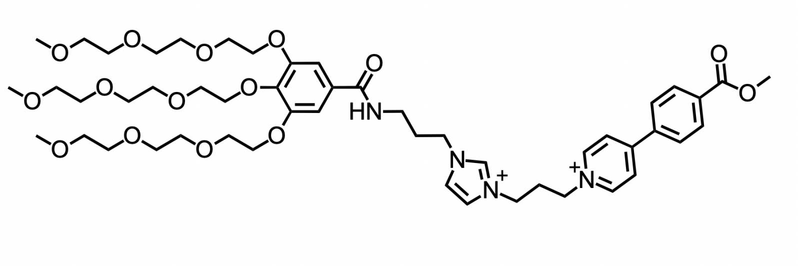

Figure S5 : Molecular structure of TEG-BPIMe. Counterions have been omitted for clarity.

TEG-I (140 mg, 0.20 mmol, 1.1 equiv) was dissolved in anhydrous CH_3CN (0.5 mL), and a solution of 1-(3-bromopropyl)-4-(4-(methoxycarbonyl)phenyl)pyridinium bromide (73.8 mg, 0.18 mmol, 1.0 equiv) dissolved in anhydrous CH_3CN (4.5 mL) was added. The reaction mixture was stirred at 60 °C for 3 days under N_2. The crude was concentrated using a rotary evaporator and further purified by preparative TLC (20% CH_3OH in CH_2Cl_2) to afford the product as colourless oil (150 mg, 75%).

^{1}H NMR (700 MHz, MeOD) δ 9.18 (d, J = 6.4 Hz, 2H), 8.51 (d, J = 6.5 Hz, 2H), 8.25 (d, J = 8.1 Hz, 2H), 8.15 (d, J = 8.2 Hz, 2H), 7.80 (s, 1H), 7.79 (s, 1H), 7.28 (s, 2H), 5.49 (s, 12H), 4.50 (t, J = 7.4 Hz, 2H), 4.38 (t, J = 6.8 Hz, 2H), 4.29 (t, J = 4.5 Hz, 4H), 4.25 (t, J = 4.5 Hz, 2H), 3.97 (s, 3H), 3.89–3.86 (m, 4H), 3.73 (dd, J = 9.6, 4.2 Hz, 4H), 3.71 (d, J = 4.7 Hz, 4H), 3.69–3.60 (m, 13H), 3.54 (ddd, J = 13.4, 5.5, 3.3 Hz, 7H), 3.50–3.43 (m, 2H), 2.72 (dd, J = 15.1, 7.5 Hz, 2H), 2.30 (t, J = 6.6 Hz, 1H).

^{13}C NMR (176 MHz, MeOD) δ 167.90, 166.06, 155.61, 151.98, 145.01, 138.14, 133.01, 130.31, 129.63, 128.26, 125.65, 122.65, 122.51, 106.23, 72.22, 71.39, 71.34, 69.96, 69.93, 69.77, 69.75, 69.74, 69.69, 69.51, 68.98, 68.42, 57.83, 57.80, 57.48, 53.44, 53.43, 51.68, 48.14, 48.02, 47.98, 47.86, 47.74, 47.62, 47.49, 47.37, 47.25, 46.16, 36.18, 31.06, 29.25 (peaks missing due to signal overlapping).

HRMS for {TEG-BPIMe^{2+}}: found m/z = 485.2572, calculated m/z = 485.2570.

2 .1.5 TEG-BPI

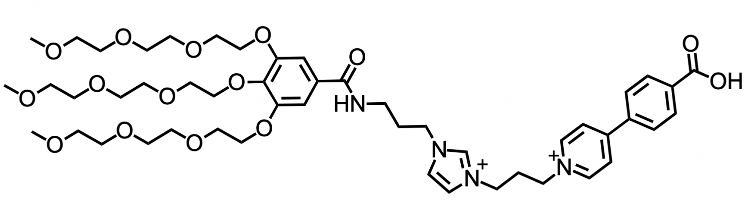

Figure S6 : Molecular structure of TEG-BPI. Counterions have been omitted for clarity.

TEG-BPIMe (20 mg, 0.018 mmol, 1.0 equiv) was added to 6 M HCl (1 mL) and was heated at 80 °C for overnight. The solvent was removed in vacuo to afford the product as a pale yellow solid (20 mg, quant.).

^{1}H NMR (500 MHz, D_2O) δ 8.79 (s, 1H), 8.72 (d, J = 6.8 Hz, 2H), 8.30 (d, J = 6.0 Hz, 2H), 8.11 (d, J = 7.9 Hz, 2H), 8.00–7.95 (m, 2H), 7.56 (s, 1H), 7.52 (s, 1H), 6.83 (s, 2H), 4.56 (q, J = 7.7, 7.2 Hz, 2H), 4.38–4.18 (m, 7H), 4.07 (dt, J = 13.6, 3.8 Hz, 6H), 3.77 (dd, J = 5.5, 3.1 Hz, 5H), 3.69–3.42 (m, 27H), 3.33 (t, J = 6.3 Hz, 2H), 3.21 (s, 5H), 2.31 (d, J = 8.0 Hz, 2H), 2.14–2.08 (m, 2H).

^{13}C NMR (126 MHz, D_2O) δ 155.78, 152.36, 144.17, 139.81, 138.05, 135.76, 132.79, 130.69, 128.38, 128.29, 126.44, 125.89, 122.86, 122.46, 106.16, 71.94, 71.68, 70.94, 70.14, 69.88, 69.82, 69.77, 69.61, 69.56, 69.50, 69.40, 69.14, 69.11, 68.33, 60.30, 57.96, 49.52, 48.21, 46.32, 37.82, 30.48, 28.17 (some signals overlapping).

HRMS for {TEG-BPI^{2+}}: found m/z = 478.2518, calculated m/z =478.2497.

2 .2 VBPI Synthesis

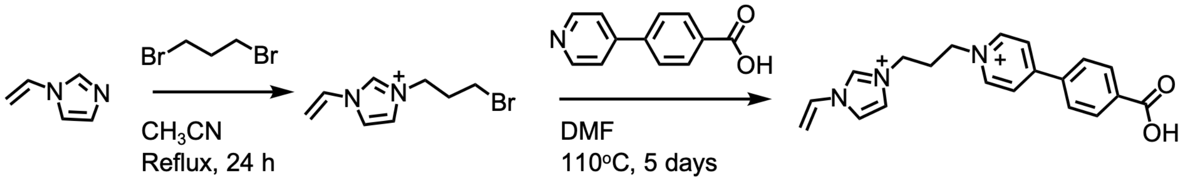

Figure S7 : Synthetic route for the preparation of VBPI. Counterions have been omitted for clarity.

2 .2.1 3-(3-bromopropyl)-1-vinyl-1H-imidazol-3-ium bromide, VI

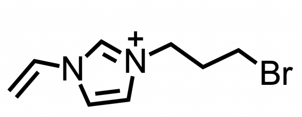

Figure S8 : Molecular structure of VI. Counterions have been omitted for clarity.

N-vinylimidazole (0.942 g, 10 mmol) and 1,3-dibromopropane (101 g, 500 mmol) were added to acetonitrile (30 ml) and refluxed overnight. The resulting mixture was transferred to four centrifuge tubes, and ether was added (30 ml each), resulting in 3-(3-bromopropyl)-1-vinyl-1H-imidazol-3-ium bromide precipitation. The intermediate was collected as the precipitate by centrifugation at 10,000 rpm for 10 minutes, washed with ether (re-suspended in 40 ml ether, and centrifuged again to collect the precipitate), and dried *in vacuo* as a yellow oil (2.8 g, 95%).

^{1}H NMR (700 MHz, D_2O) δ 9.06 (t, J = 1.7 Hz, 1H), 7.74 (t, J = 1.9 Hz, 1H), 7.57 (t, J = 1.9 Hz, 1H), 7.09 (dd, $J = 15.6, 8.7$ Hz, 1H), 5.75 (dd, $J = 15.6, 2.9$ Hz, 1H), 5.38 (dd, $J = 8.6, 2.8$ Hz, 1H), 4.39 (t, $J = 6.8$ Hz, 2H), 3.42 (t, $J = 6.2$ Hz, 2H), 2.40 (p, $J = 6.6$ Hz, 2H).

^{13}C NMR (176 MHz, D_2O) δ 134.78, 128.20, 122.94, 119.76, 109.57, 48.08, 31.54, 29.28.

HRMS for {VI}: found m/z = 215.0178, calculated m/z = 215.0178.

2 .2.2 4-(4-carboxyphenyl)-1-(3-(1-vinyl-1H-imidazol-3-ium-3-yl)propyl)pyridin-1-ium dibromide, VBPI

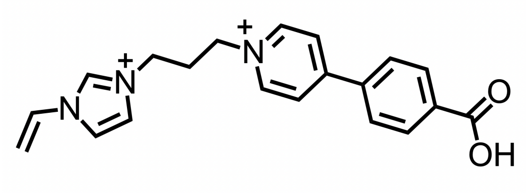

Figure S9 : Molecular structure of VBPI. Counterions have been omitted for clarity.

3-(3-bromopropyl)-1-vinyl-1H-imidazol-3-ium bromide (2.8 g, 9.5 mmol, 95% yield) and 4-(pyridin-4-yl)benzoic acid (0.6 g, 3 mmol) were added to a mixture of *N,N*-dimethylformamide (75 ml) and acetonitrile (75 ml) and refluxed for 5 days. The resulting mixture was transferred to four centrifuge tubes and precipitated with acetonitrile (20 ml each), resulting in 4-(4-carboxyphenyl)-1-(3-(1-vinyl-1H-imidazol-3-ium-3-yl)propyl)pyridin-1-ium dibromide precipitation as a white crystalline solid. The white solid was collected as the precipitate by centrifugation at 10,000 rpm for 10 minutes, washed with ether (re-suspended in 40 ml ether, and centrifuged again to collect the precipitate), and dried *in vacuo* to afford a white solid. (1.3 g, 87% yield).

^{1}H NMR (400 MHz, D_2O) δ 9.07 (s, 1H), 8.83 (d, J = 6.4 Hz, 2H), 8.32 (d, J = 6.4 Hz, 2H), 8.09 (d, J = 8.1 Hz, 2H), 7.95 (d, J = 8.1 Hz, 2H), 7.75 (s, 1H), 7.58 (s, 1H), 7.07 (dd, J = 15.6, 8.7 Hz, 1H), 5.37 (d, J = 8.9 Hz, 1H), 4.41 (t, J = 7.3 Hz, 2H), 2.66 (q, J = 7.4 Hz, 2H).

^{13}C NMR (126 MHz, D_2O) δ 169.68, 155.83, 144.43, 137.90, 134.75, 133.01, 130.56, 128.26, 128.08, 125.85, 122.80, 119.97, 109.86, 57.75, 46.58, 30.53.

HRMS for {$VBPI^{2+}$}: found m/z = 167.5802, calculated m/z = 167.5812.

2 .3 MeVBPI Synthesis

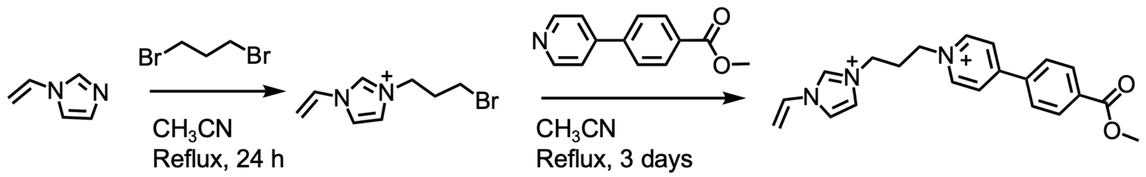

Figure S10 : Synthetic route for the preparation of MeVBPI. Counterions have been omitted for clarity.

3-(3-bromopropyl)-1-vinyl-1H-imidazol-3-ium bromide (2.8 g, 9.5 mmol, 95% yield) and methyl 4-(pyridin-4-yl)benzoate (2.2 g, 10.45 mmol) were added to acetonitrile (30 ml) and refluxed for 72 h. The resulting mixture was transferred to two centrifuge tubes and precipitated with ether (30 ml each), resulting in 4-(4-(methoxycarbonyl)phenyl)-1-(3-(1-vinyl-1H-imidazol-3-ium-3-yl)propyl)pyridin-1-ium dibromide precipitation as a white crystalline solid. The white solid was collected as the precipitate by centrifugation at 10,000 rpm for 10 minutes, washed with ether (re-suspended in 40 ml ether, and centrifuged again to collect the precipitate), and dried *in vacuo* to afford a white solid. (3.36 g, 69% yield).

^{1}H NMR (400 MHz, D_2O) δ 8.85 (d, J = 6.9 Hz, 2H), 8.37 – 8.30 (m, 2H), 8.20 – 8.13 (m, 2H), 8.00 – 7.93 (m, 2H), 7.75 (d, J = 2.2 Hz, 1H), 7.59 (d, J = 2.2 Hz, 1H), 7.07 (dd, $J = 15.5, 8.7$ Hz, 1H), 5.73 (dd, $J = 15.6, 2.9$ Hz, 1H), 5.37 (dd, $J = 8.7, 2.9$ Hz, 1H), 4.41 (t, J = 7.3 Hz, 2H), 3.91 (s, 3H), 2.67 (p, J = 7.5 Hz, 2H).

^{13}C NMR (126 MHz, D_2O) δ 168.33, 155.90, 144.47, 138.12, 134.76, 132.38, 130.41, 128.31, 128.09, 125.93, 122.80, 119.97, 109.86, 57.77, 52.93, 46.58, 30.54.

HRMS for {$MeVBPI^{2+}$}: found m/z = 174.5925, calculated m/z = 174.5895.

3 Spectral Characterisation of Previously Unreported Molecules

3 .0.1 Stopper NHS ester, TEG-NHS

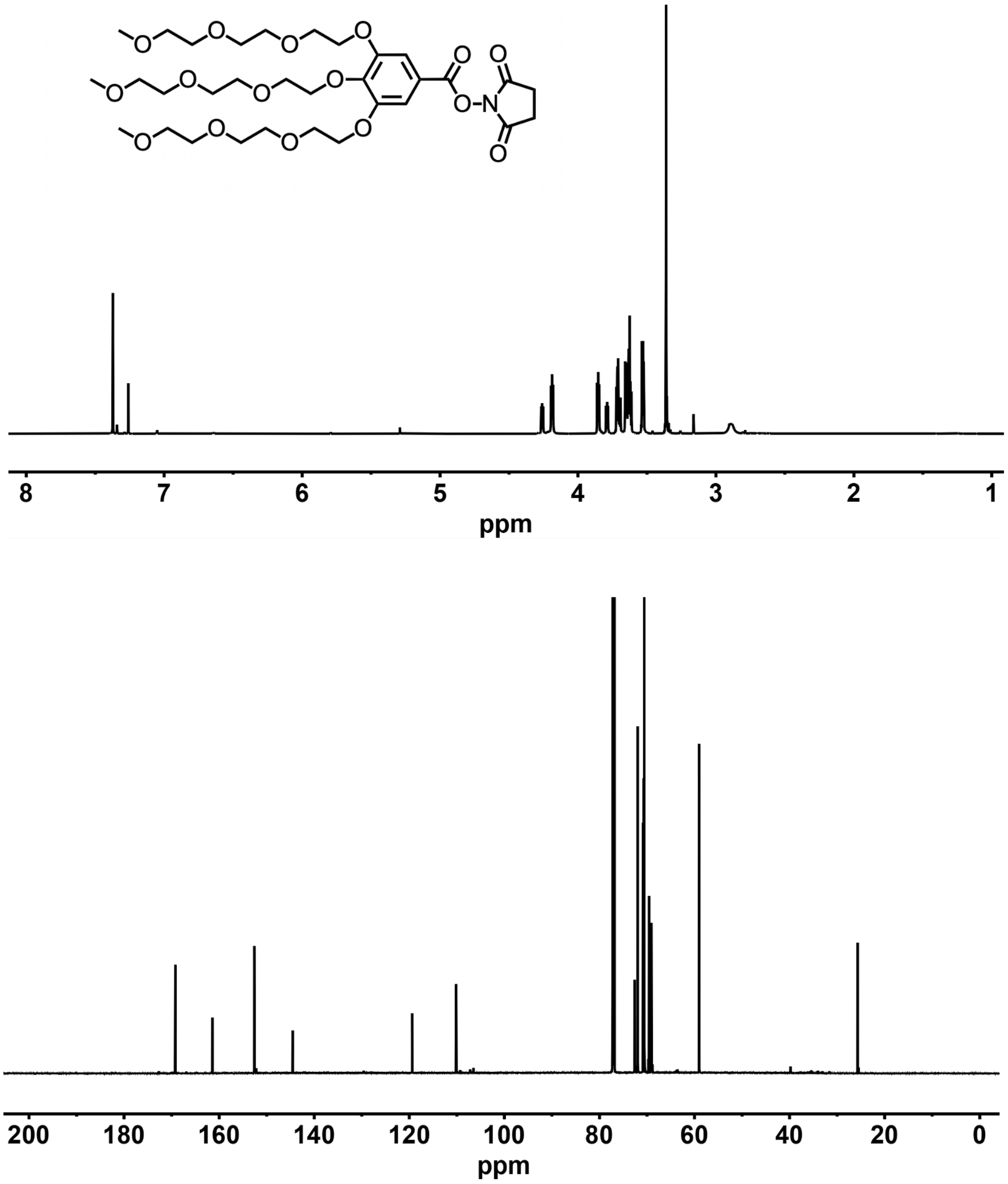

Figure S11 : ^{1}H NMR spectra ($CDCL_3$, 298 K) for TEG-NHS (above). ^{13}C NMR spectra ($CDCL_3$, 298 K) for TEG-NHS (below).

3 .0.2 Stopper imidazole, TEG-I

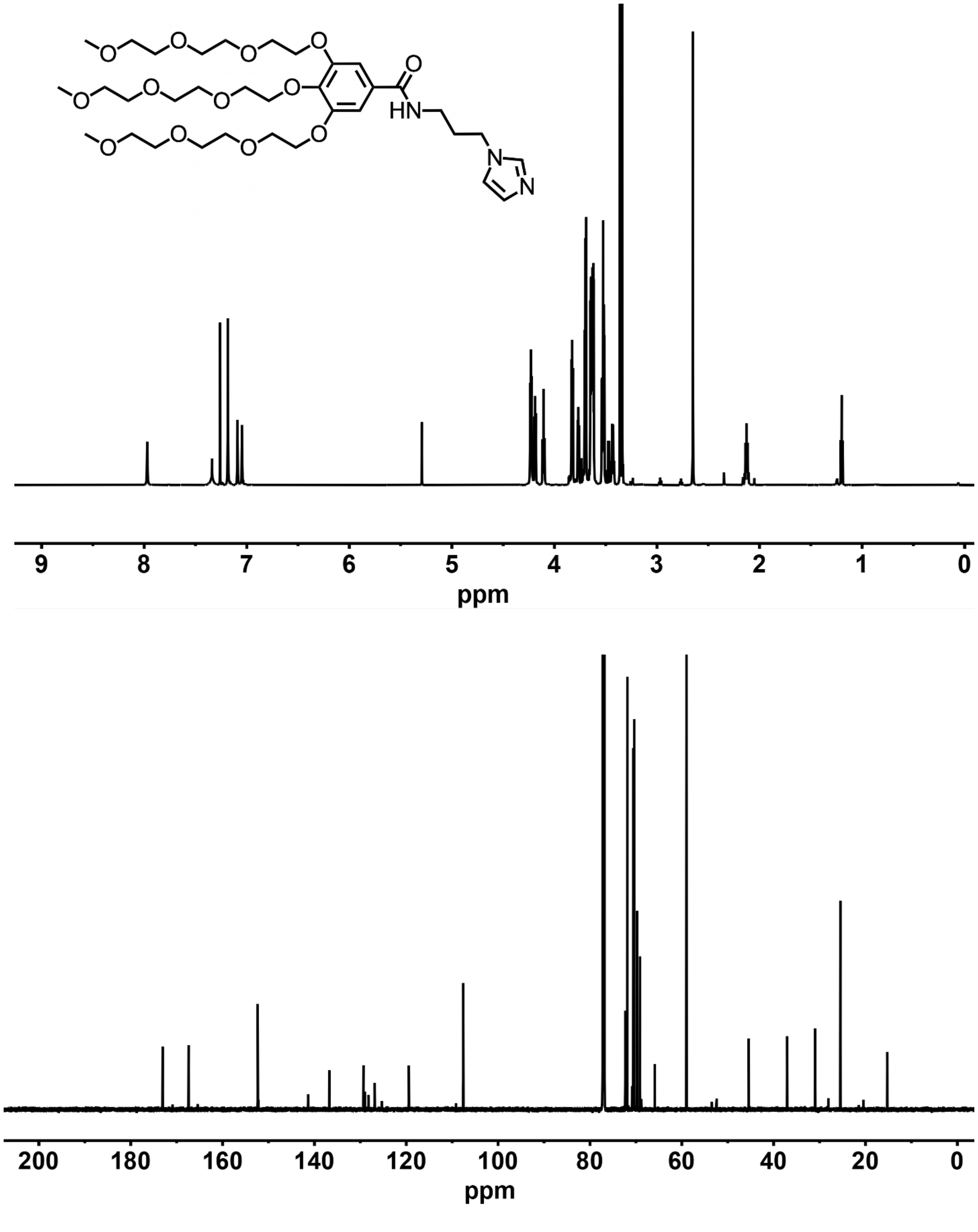

Figure S12 : ^{1}H NMR spectra ($CDCL_3$, 298 K) of TEG-I (above). ^{13}C NMR spectra ($CDCL_3$, 298 K) of TEG-I (below).

3 .0.3 1-(3-bromopropyl)-4-(4-(methoxycarbonyl)phenyl)pyridinium bromide, BPMe

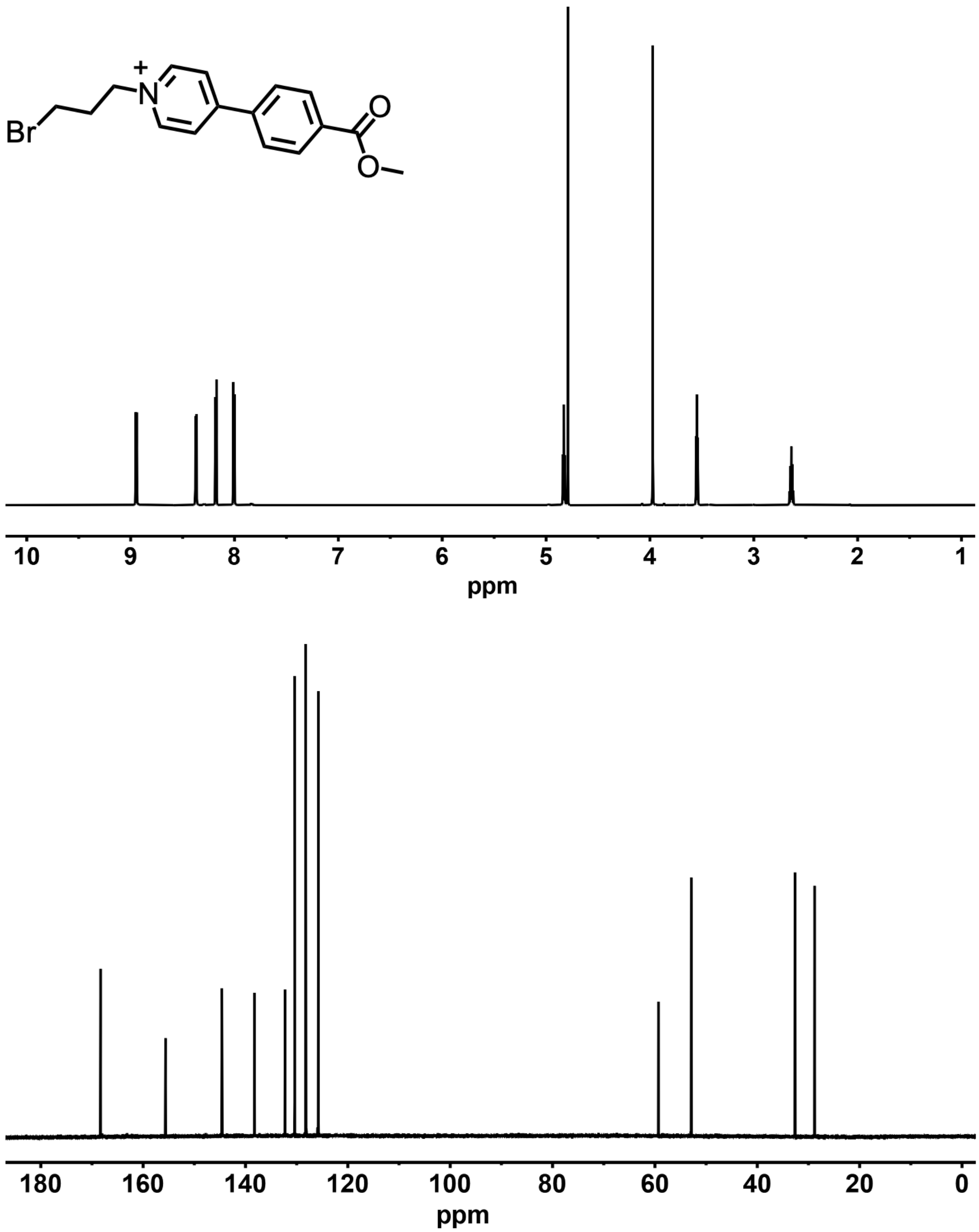

Figure S13 : ^{1}H NMR spectra (D_2O, 298 K) of BPMe (above). ^{13}C NMR spectra (D_2O, 298 K) of BPMe (below).

3 .0.4 TEG-BPIMe

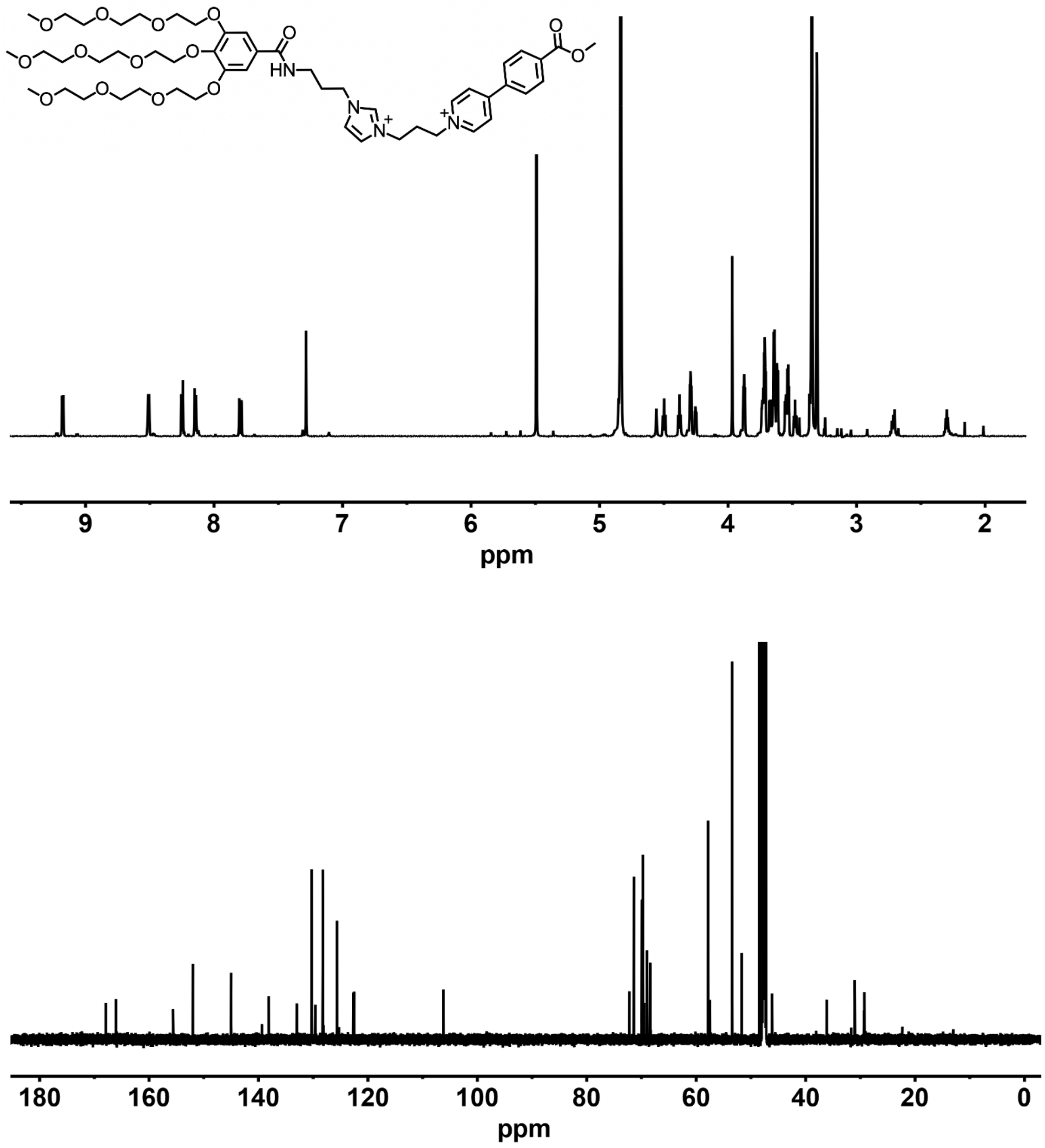

Figure S14 : ^{1}H NMR spectra (MeOD, 298 K) of TEG-BPIMe (above). ^{13}C NMR spectra (MeOD, 298 K) of TEG-BPIMe (below).

3 .0.5 TEG-BPI

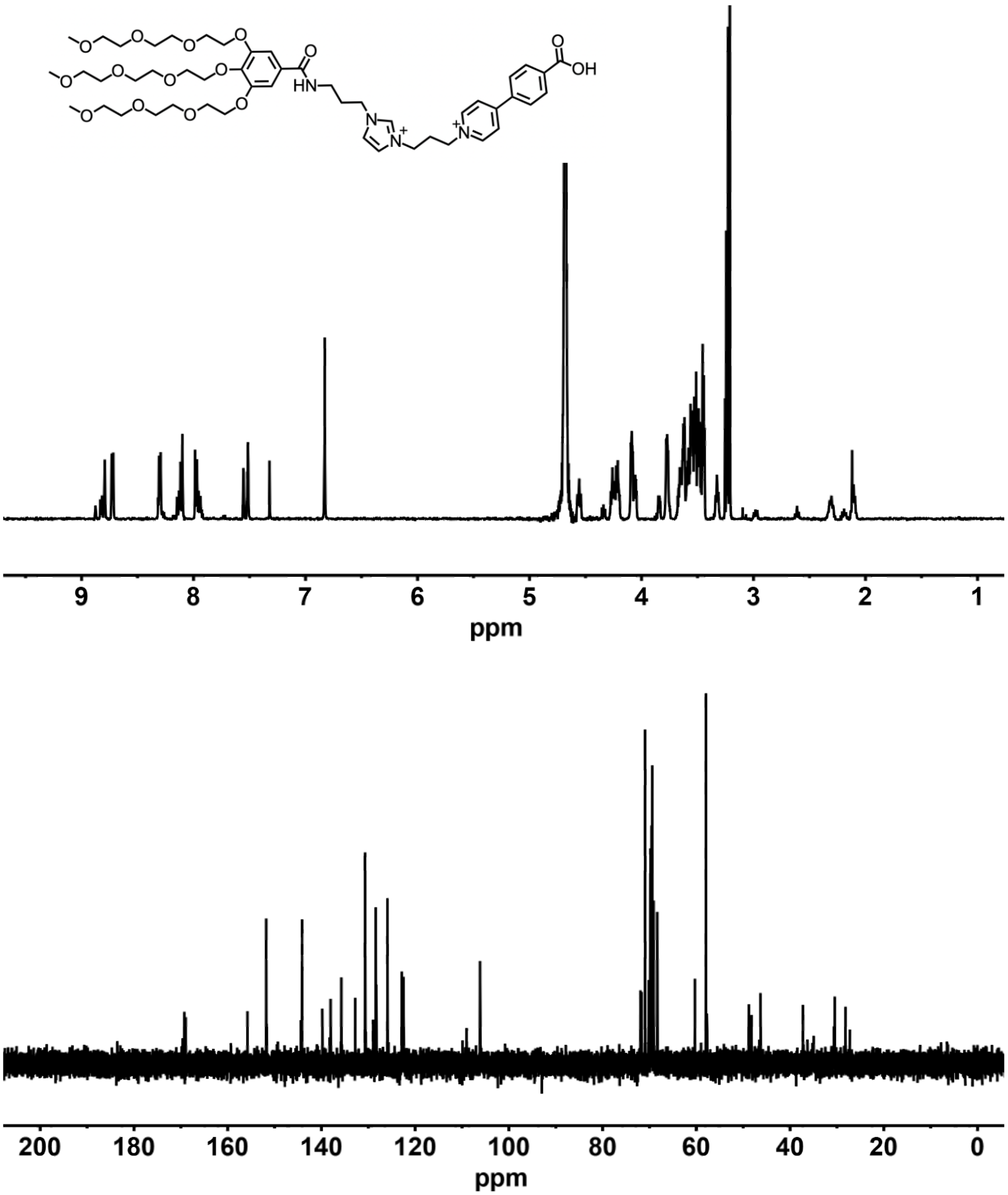

Figure S15 : ¹H NMR spectra (MeOD, 298 K) of TEG-BPI (above). ¹³C NMR spectra (MeOD, 298 K) of TEG-BPI (below).

3 .0.6 3-(3-bromopropyl)-1-vinyl-1H-imidazol-3-ium bromide, VI

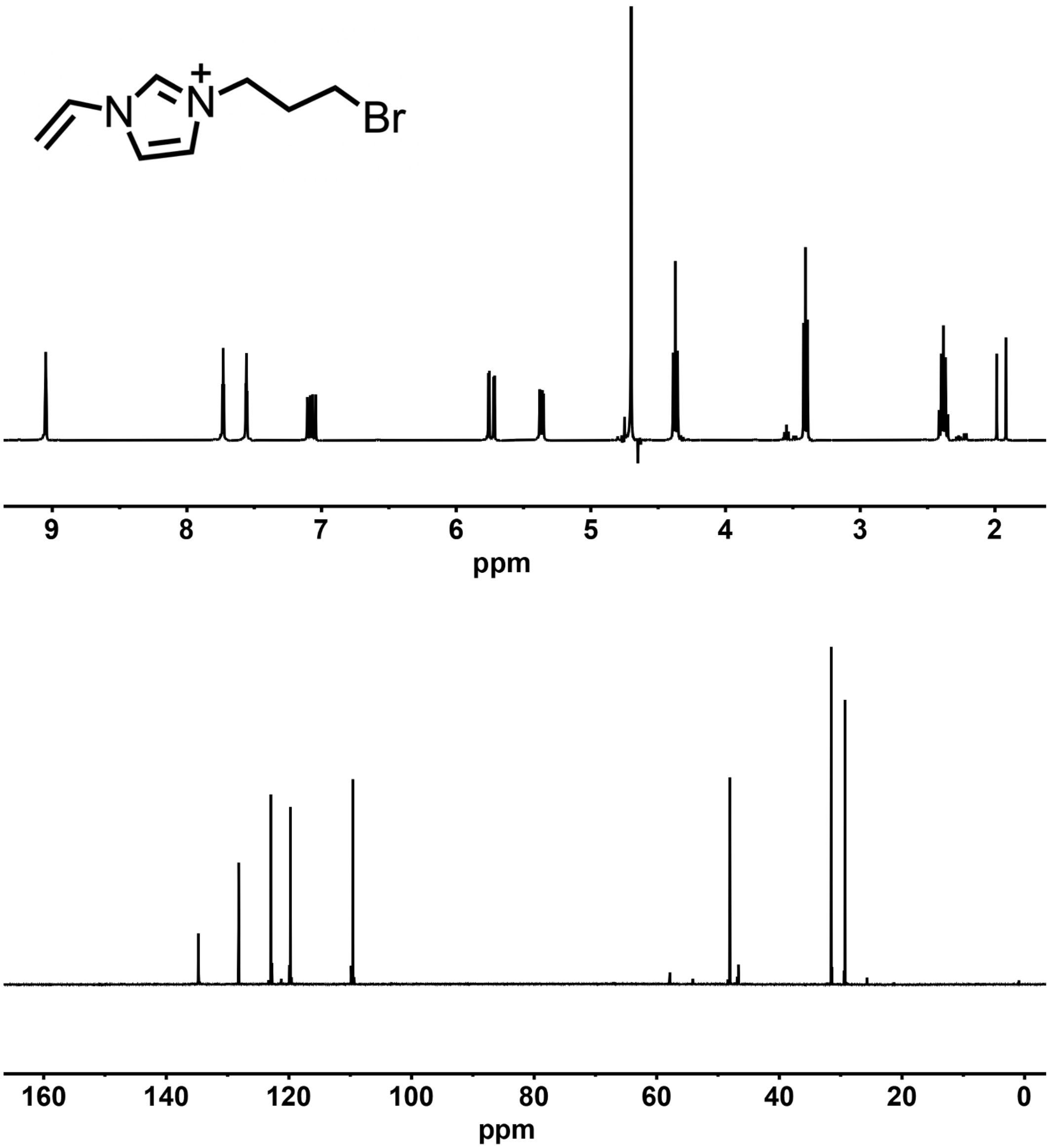

Figure S16 : ^{1}H NMR spectra (D_2O, 298 K) of VI (above). ^{13}C NMR spectra (D_2O, 298 K) of VI (below).

3 .0.7 VBPI

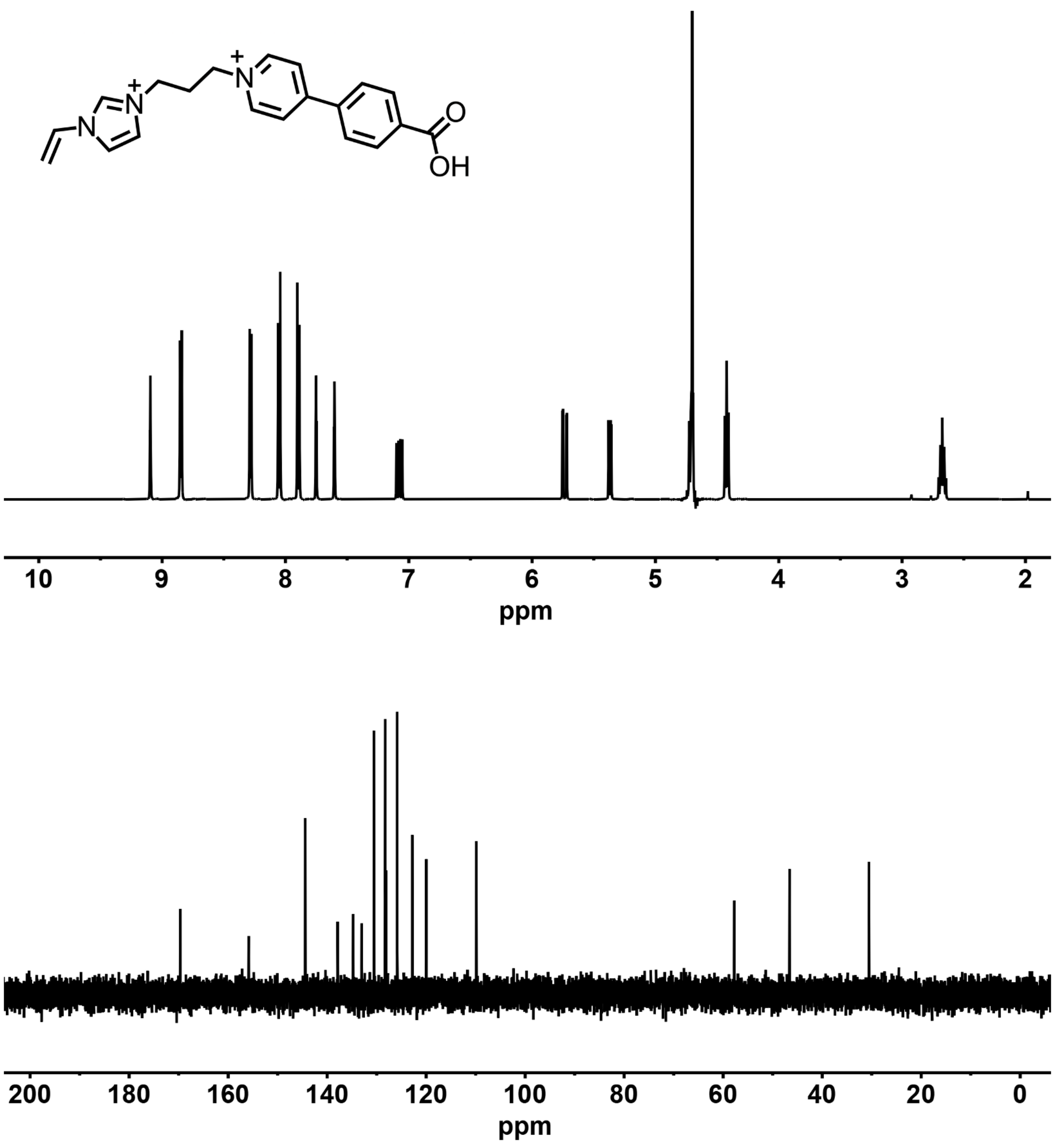

Figure S17 : ^{1}H NMR spectra (D_2O, 298 K) of VBPI (above). ^{13}C NMR spectra (D_2O, 298 K) of VBPI (below).

3 .0.8 MeVBPI

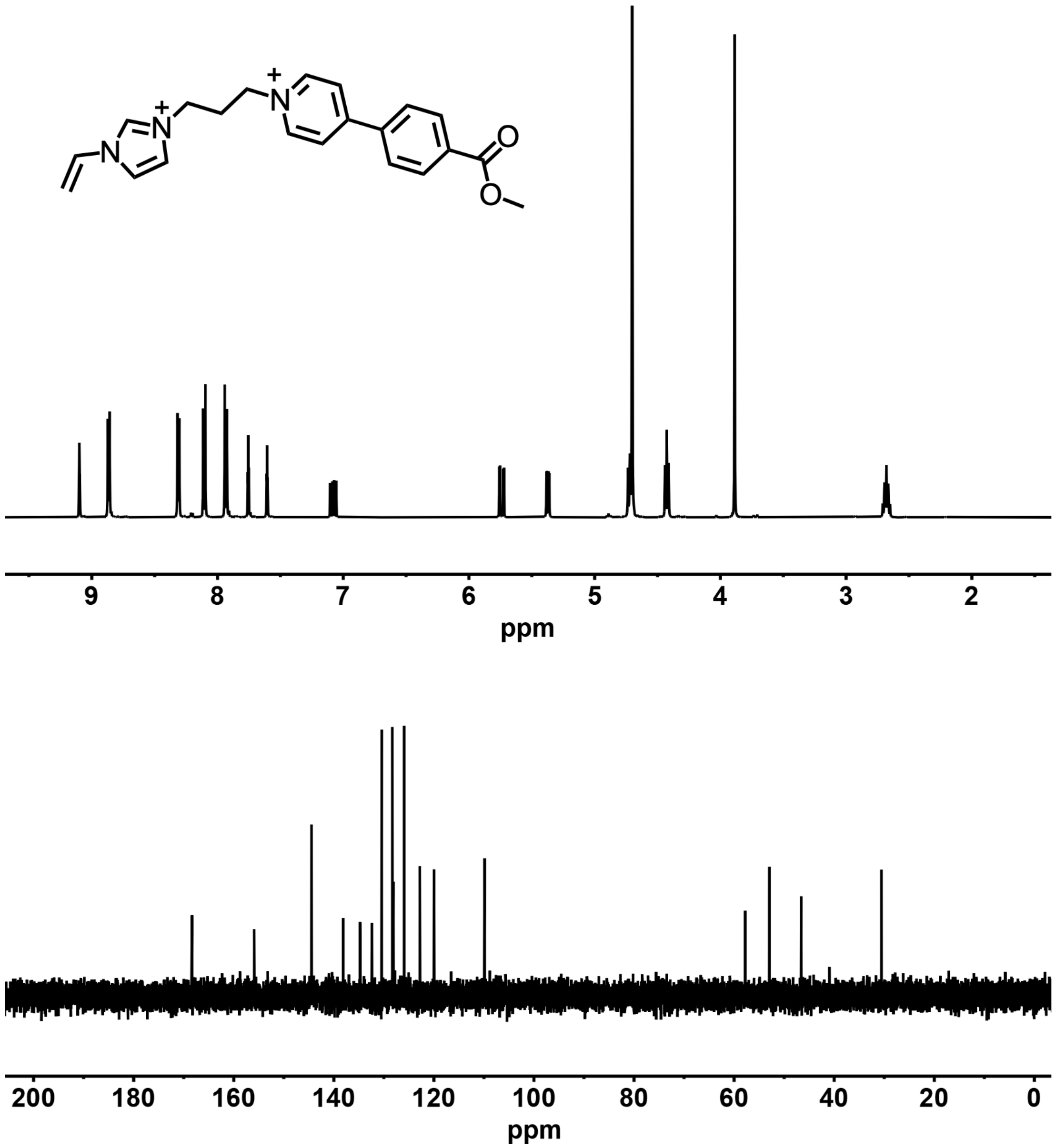

Figure S18 : ^{1}H NMR spectra (D_2O, 298 K) of MeVBPI (above). ^{13}C NMR spectra (D_2O, 298 K) of MeVBPI (below).

4 Binding Studies

4 .1 NMR titration of CB[7] to TEG-BPI

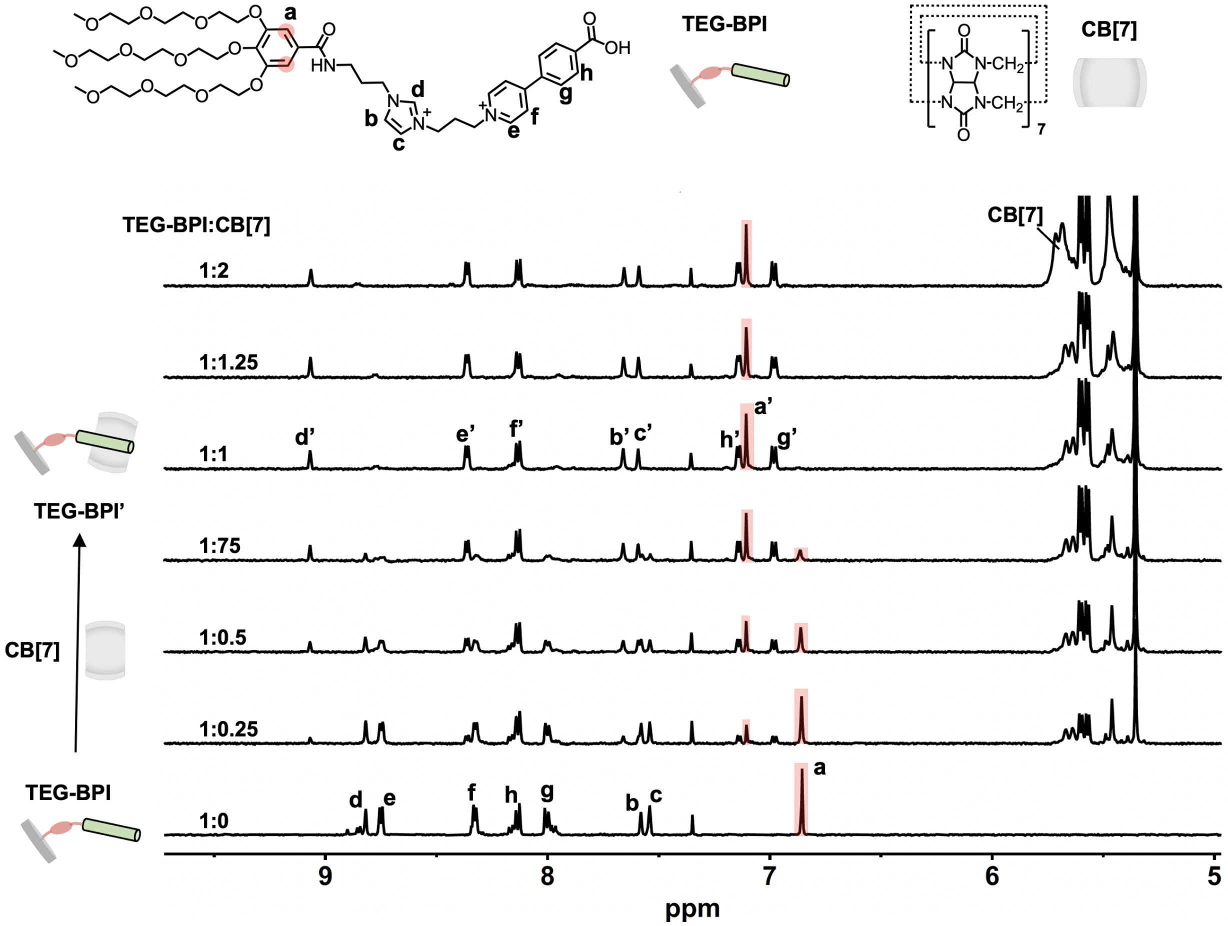

Figure S19 : Titration of CB[7] into TEG-BPI, monitoring of ^{1}H NMR shifts of protons on TEG-BPI. ^{1}H NMR spectra (D_2O, 298 K) given for TEG-BPI guest alone (bottom), titrating until the ratio of TEG-BPI:CB[7] equals 1:2 (top). Counterions have been omitted for clarity.

4 .2 Ion-dipole repulsion investigation

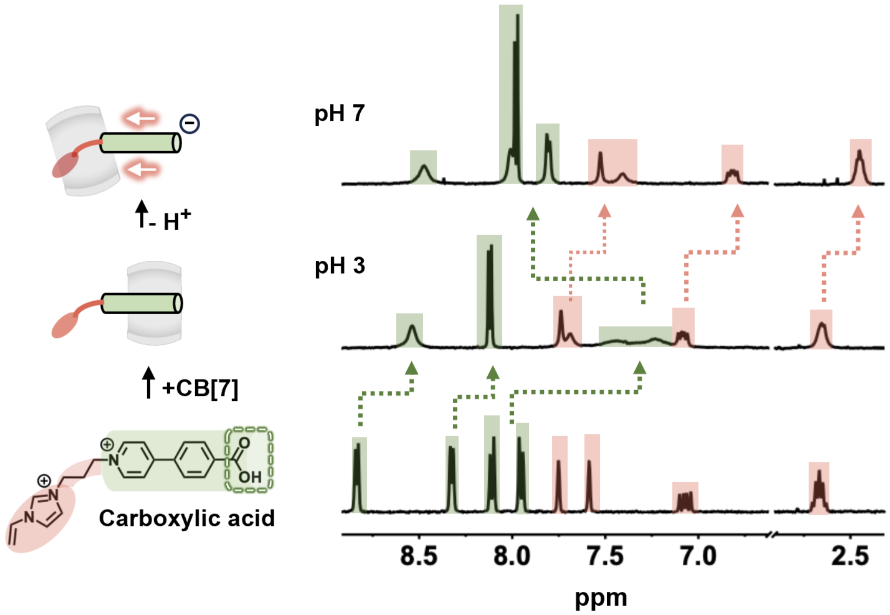

Figure S20 : ^{1}H NMR spectra (D_2O, 298 K) of VBPI guest alone (bottom), VBPI·CB[7] complex at pH 3 (middle) and VBPI-CB[7] complex at pH 7 (top). Counterions have been omitted for clarity.

The effect of protonation and deprotonation on binding were investigated through ^{1}H NMR, Figure S20 . The smaller analogue CB[7] was employed, which binds 1:1 with VBPI and therefore allows easier observation of the position of the CB molecule through changes in proton environments of the guest. In the protonated form at pH 3, upfield shifts are observed for the phenyl and pyridine groups (highlighted in green) suggesting encapsulation with the CB[7] cavity. It was found that when the guest becomes deprotonated at pH 7, the protons associated with the phenyl and pyridine groups shift back downfield, while the protons associated with the methylene and imidazole groups (highlighted in blue) shift upfield. Such observations suggest ion-dipole repulsion (on account of deprotonation of the carboxylic acid) causes the CB[7] to migrate towards the imidazolium, methylene and vinyl groups.

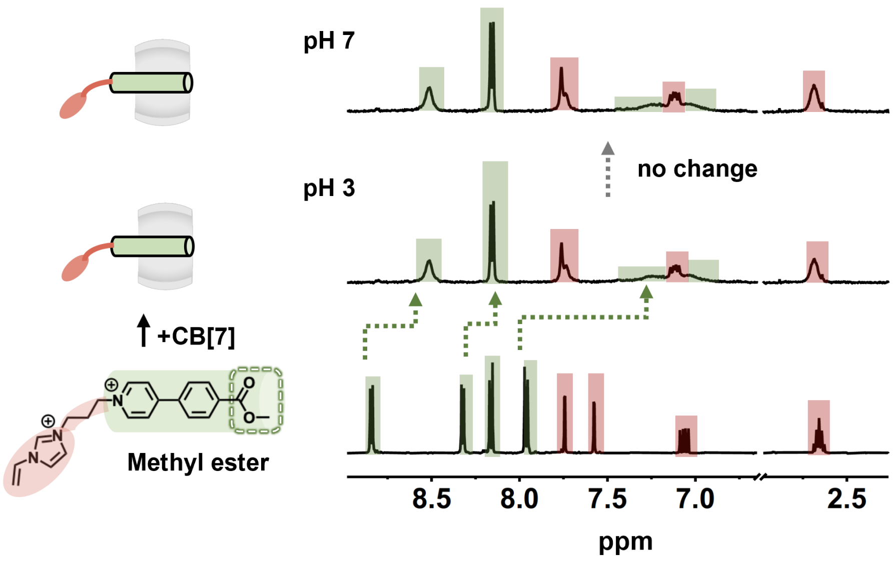

Figure S21 : ^{1}H NMR spectra (D_2O, 298 K) of MeVBPI guest alone (bottom), MeVBPI·CB[7] complex at pH 3 (middle) and MeVBPI·CB[7] complex at pH 7 (top). Counterions have been omitted for clarity.

A control guest molecule was synthesised using a methyl ester instead of a carboxylic acid, giving 4-(4-(methoxycarbonyl)phenyl)-1-(3-(1-vinyl1H-imidazol-3-ium-3yl)propyl)-pyridin-1-ium (MeVBPI), Figure S28 . No deprotonation or anion formation would occur with a methyl ester under the working pH range. Indeed, the complex was unaffected when switching between pH 3 and pH 7. Under both acidic and neutral conditions, the protons associated with phenyl and pyridine groups remained shifted upfield, suggesting the interaction with CB[7] is pH independent. Such findings confirm the role of the carboxylic acid moiety towards pH-dependent binding of VBPI with CB[7].

4 .3 pH-dependent binding investigation

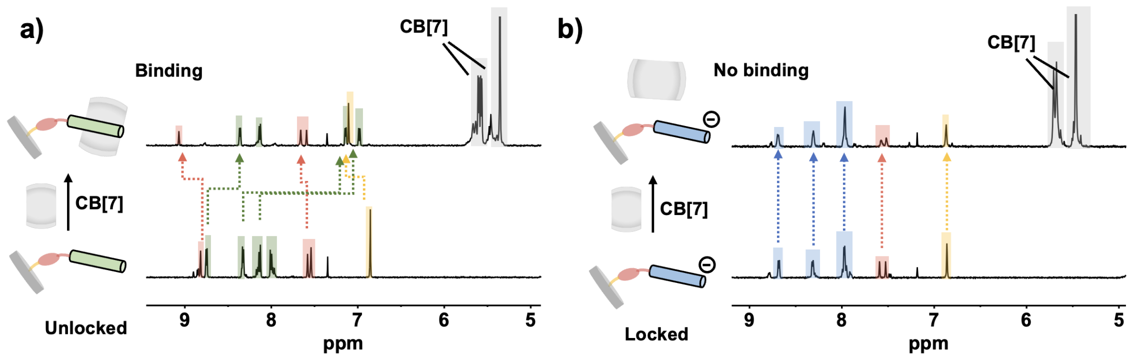

Figure S22 : a) ^{1}H NMR spectra (D_2O, 298 K) of TEG-BPI alone (bottom), and after the introduction of excess CB[7] (top), under acidic conditions (pH = 3). b) ^{1}H NMR spectra (D_2O, 298 K) of TEG-BPI^{-} alone (bottom), and after the introduction of excess CB[7] (top), under basic conditions (pH = 8). Counterions have been omitted for clarity.

To probe whether there is any threading in the locked state, CB[7] was added to the deprotonated guest and ^{1}H NMR studies were performed, Figure S22. In the unlocked state, clear binding is observed with upfield shifting for the phenyl and pyridine groups (highlighted in green) suggesting encapsulation within the CB[7] cavity, Figure S22a. Meanwhile in the locked state, no binding was observed, with the protons for the guest molecule remaining at shift values corresponding to the free guest without CB[7], Figure S22b. Meanwhile

4 .4 Time dependent NMR

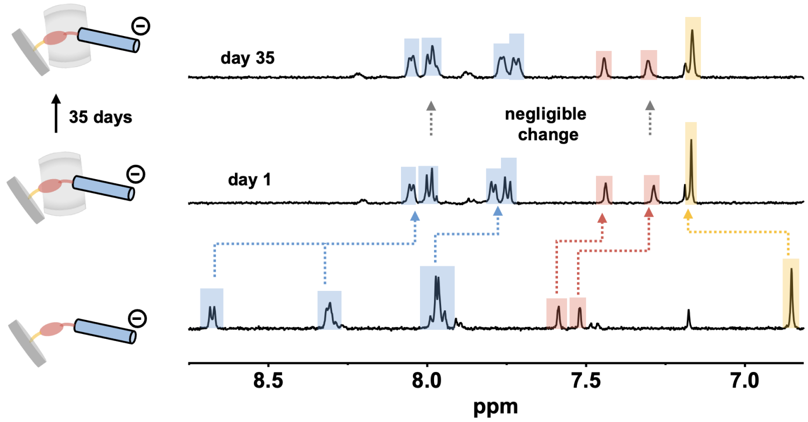

Figure S23 : ^{1}H NMR spectra (D_2O, 298 K) under basic conditions (pH = 8) of TEG-BPI alone (bottom), TEG-BPI·CB[7] after initial introduction of CB[7] (middle), and TEG-BPI·CB[7] after 35 days (top). Counterions have been omitted for clarity.

To further investigate the stability of the locked complex, ^{1}H NMR studies were performed to probe whether de-threading could occur over long periods of time, Figure S23. Over a period of 35 days at room temperature, negligible change was observed in the ^{1}H NMR spectra of the locked complex, with the guest peaks remaining at shift values corresponding to the bound state.

4 .5 Binding Investigation by HR ESI-MS

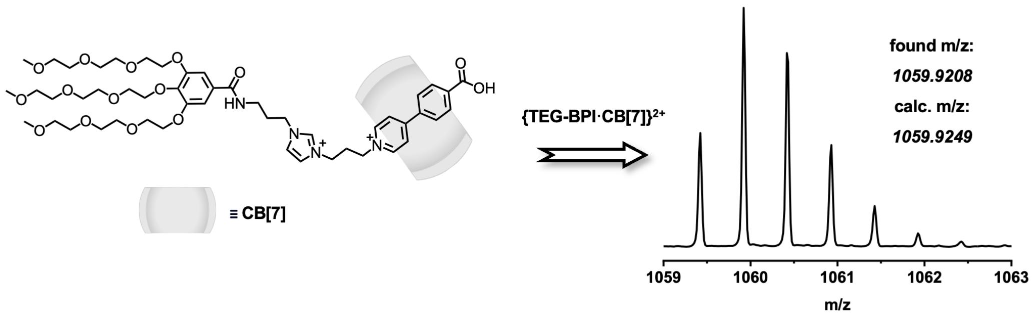

Figure S24 : HR ESI-MS spectrum (from H_2O, 0.1 mM) of TEG-BPI·CB[7] complex.

High resolution electrospray ionisation mass spectrometry (HR ESI-MS) experiments were conducted to obtain the ion peaks of the host-guest complex for the BPI-derivative TEG-BPI·CB[7], Figure S24 . Experimental m/z values consistent with the calculated values was found for the 1:1 TEG-BPI·CB[7] complex.

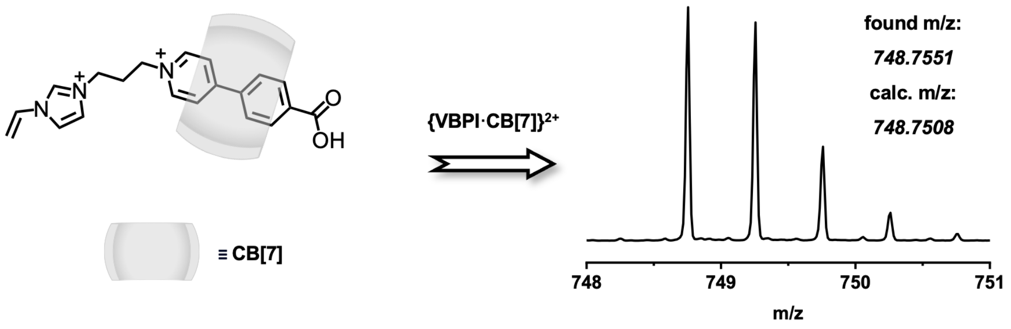

Figure S25 : HR ESI-MS spectrum (from H_2O, 0.1 mM) of TEG-BPI·CB[7] complex.

HR ESI-MS experiments were also conducted to obtain the ion peaks of the host-guest complex for the VBPI-derivatives, Figure S25 . Experimental m/z values consistent with the calculated values was found for the 1:1 VBPI·CB[7] complex.

4 .6 Binding Studies by ITC

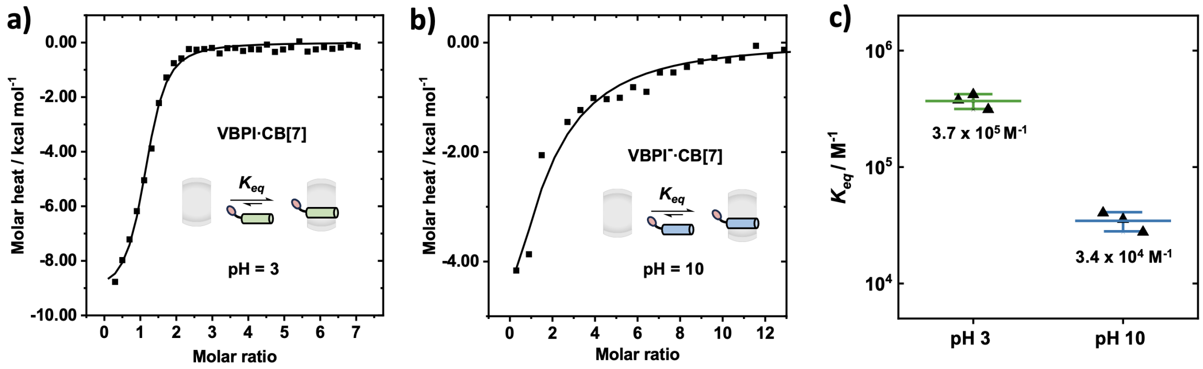

Figure S26 : a) Representative ITC (H_2O, 298 K) titration curve obtained by titrating VBPI into CB[7] at pH 3. b) Representative ITC (H_2O, 298 K) titration curve obtained by titrating VBPI into CB[7] at pH 10. c) Calculated K_{eq} values for for VBPI in pH 3 and pH 10 environments. All ITC experiments were repeated 3 times, with the average K_{eq} value given.

In the case of CB[7], there is a clear drop in the binding strength with VBPI in the basic environment, by over an order of magnitude from K_{eq} = 3.7 x 10^5 M^{-1} at pH 3 to K_{eq} = 3.4 x 10^4 M^{-1} at pH 10 (Figure S26). The drop in binding strength can be attributed to the anion formation at the guest terminal and ion-dipole repulsion. In the basic state, the anion restricts the CB[7] from binding in its preferred position on the guest, as also observed by NMR (Figure S20).

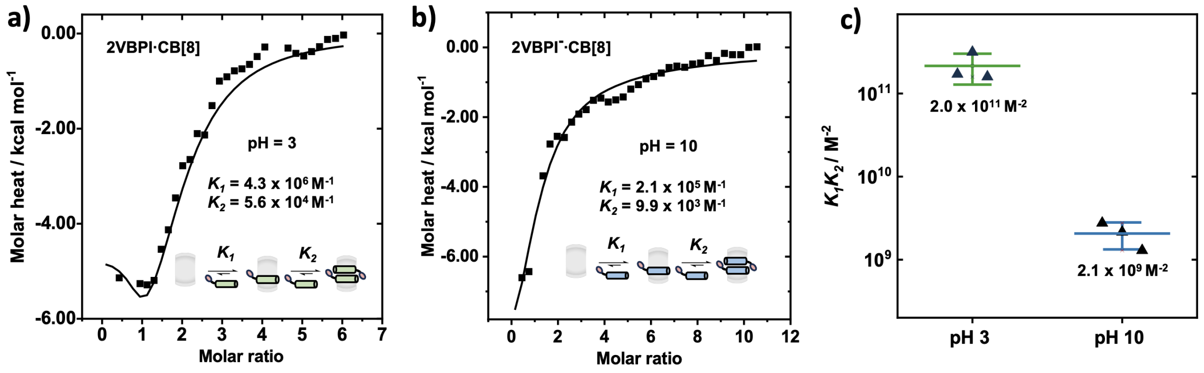

Figure S27 : a) Representative ITC (H_2O, 298 K) titration curve obtained by titrating VBPI into CB[8] at pH 3. b) Representative ITC (H_2O, 298 K) titration curve obtained by titrating VBPI into CB[8] at pH 10. c) Calculated overall binding strength (K_1K_2) values for for VBPI in pH 3 and pH 10 environments. All ITC experiments were repeated 3 times, with the average K_{eq} values given.

Similarly, in the case of CB[8], a clear decrease in both K_1 and K_2 binding constants is observed in basic environments. The K_1 value drops from 4.3 x 10^6 M^{-1} at pH 3 to 2.1 x 10^5 M^{-1} at pH 10, while the K_2 value drops from 5.6 x 10^4 M^{-1} at pH 3 to 9.9 x 10^3 M^{-1} at pH 10 (Figure S27). The overall binding strength (K_1K_2) of the ternary complex therefore drops by two orders of magnitude, from 2.0 x 10^{11} M^{-2} at pH 3 to 2.1 x 10^9 M^{-2} at pH 10 upon formation of the anion on the guest, and subsequent ion-dipole repulsion affects.

5 Variable temperature investigation

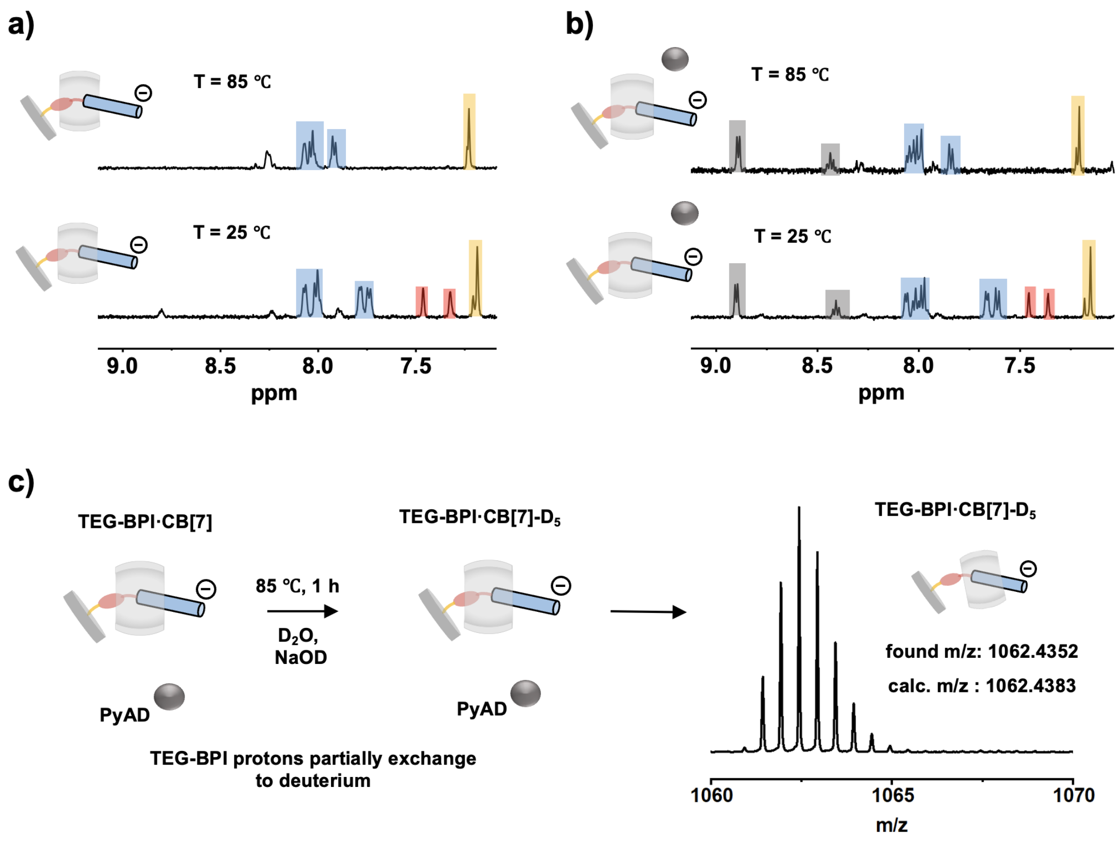

Figure S28 : a) ^{1}H NMR spectra (D_2O) of TEG-BPI^{-}·CB[7] at 25 °C (bottom) and 85 °C (top). b) ^{1}H NMR spectra (D_2O) of TEG-BPI^{-}·CB[7] in the presence of PyAD competitive guest at 25 °C (bottom) and 85 °C (top). c) HR ESI-MS spectrum (from D_2O, 0.1 mM) of TEG-BPI·CB[7] in the presence of PyAD competitive guest after 1 h at 85 ^{o}C at pH = 9. Counterions have been omitted for clarity.

To probe whether there is an energy barrier for CB[7] to de-thread TEG-BPI, the locked complex was heated up to 85 °C, and ^{1}H NMR and HR-ESI MS experiments were performed (Figure S28). Once heated, it was found that the protons belonging to the pyridinium and imidazolium moieties on the guest disappear, Figure S28a. However, the protons corresponding to the phenyl moieties (yellow and blue), remain at shift values similar to the bound state. The same experiment was also performed in the presence of PyAD competitive guest, Figure S28b. Similarly, the pyridinium and imidazolium protons are missing, while the phenyl protons remain at shift values corresponding to the guest remaining bound to CB[7]. To confirm CB[7] remains bound during this experiment, the sample was kept at 85 °C for 1 h and HR ESI-MS was performed, Figure

S28c. A mass equivalent to the locked complex (with CB[7] still bound), except with an added weight corresponding to five deuterium's instead of protons, was observed. Such evidence explains the loss in signals of the pyridinium and imidazolium protons during the ^{1}H NMR experiments, on account of exchange with deuterium. These findings substantiate the stability of the locked complex, suggesting that any energy barrier for de-threading that may exist would be above 85 °C.

6 Determination of pK_a

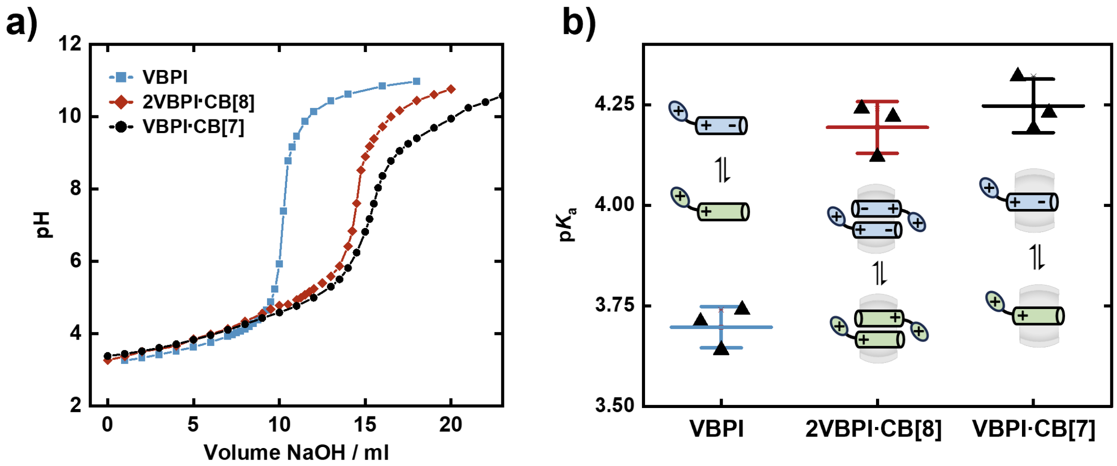

Figure S29 : a) Titration of 1 mM NaOH into solutions of both the guest alone, and host-guest complexes, at 1.0 mM. b) Comparison of pK_a values for both VBPI alone and VBPI complexes with CB[n].

The pK_a of the newly synthesised guest was determined through a pH-based titration, both for the guest alone and existing as the CB[7] 1:1 complex, as well as the CB[8] 2:1 ternary complex, Figure S29 . Aliquots of sodium hydroxide (NaOH) were added to an aqueous solution containing either the guest or the complex, and the pH was monitored. The pK_a was then calculated through taking half the pH value of the inflection point. It was found that upon complexation, the pK_a increased by over 0.5 pK_a units. Such phenomenon has previously been reported for host-guest systems, in which the binding to CB[8] retards the dissociation of a proton to a higher pH.[S5]

7 Polymer Network Formation

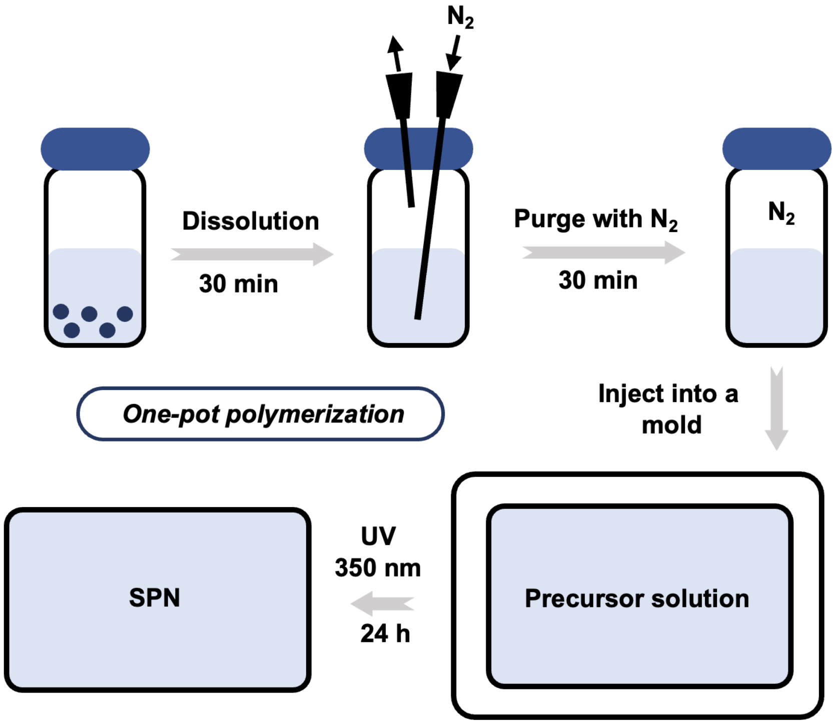

Figure S30 : Schematic representation for the preparation of supramolecular polymer networks through one-pot polymerisation in a glass mould.

Supplementary Figure S30 highlights the procedure for the preparation of polymer networks.

(1) Acrylamide (AAm), non-covalent cross-linker (2VBPI-CB[8]), and photoinitiator (irgacure 2959) were weighed out in a glass vial, and dissolved (or dispersed) in Milli-Q water under ultrasonication for 30 min. The polymer networks were formed at a concentration of 0.95 M AAm, 0.05 M VBPI, 0.025 M CB[8] and 0.001 M I-2959.

(2) The obtained precursor solution was sealed and purged with nitrogen for at least 30 min to remove oxygen in the solution that may eliminate radicals during polymerisation.

(3) The precursor solution was carefully injected into a laboratory-made glass mould until the entire mould was filled without empty space inside.

(4) The glass mould filled with the precursor solution was exposed to UV irradiation at 350 nm with 4.8 mW/cm^2 for 6 h to undergo in situ photo-polymerisation.

(5) After in situ polymerisation, the polymer networks were removed from the glass mould and further cut into the test specimens with different sizes and shapes using either a dumbbell/cylinder-shaped cutter or a razor blade.

The prepared polymer networks were directly used in subsequent characterisation or demonstration without further purification.

8 Mechanical & Rheological Characterisation

8 .1 Frequency sweep measurements

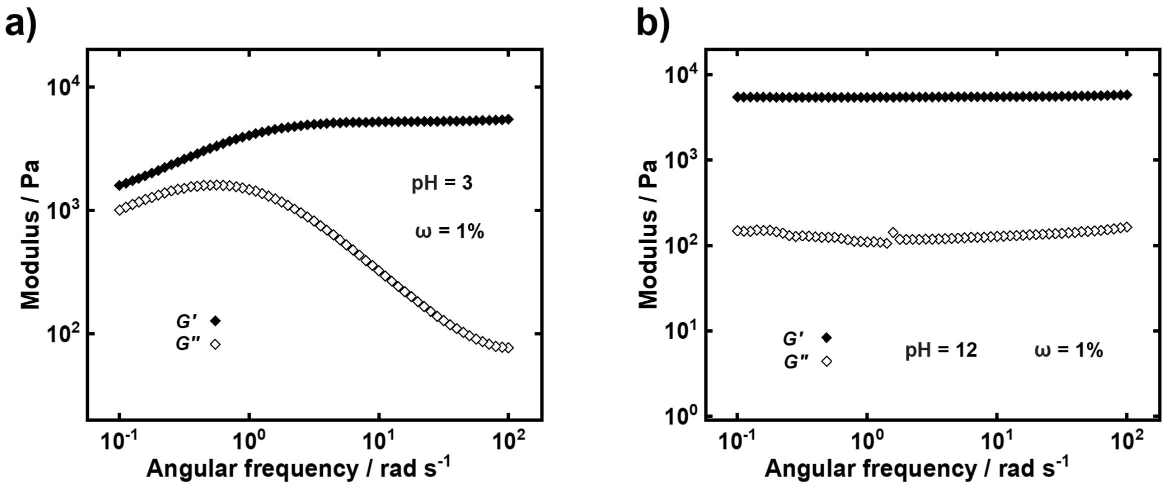

Figure S31 : Rheological frequency sweep measurements of the supramolecular polymer networks under (a) acidic (pH 3) and (b) basic (pH 12) conditions.

pH-dependent viscoelastic behaviour was observed for the SPN through frequency sweep measurements. Tan δ measurements (quantified by the ratio of loss to storage modulus) were performed across a variety of frequencies, which is a measure of a materials viscoelasticity. It is calculated by the ratio of viscoelastic to elastic forces of the material, ranging from a value of 0 to 1 for gel-state materials. A tan δ closer to zero indicates a more elastic material. Dynamic crosslinks typically exhibit a frequency dependent tan δ, varying across frequencies depending on the kinetics of the crosslinks. At lower frequencies, the material undergoes slow deformations, giving the supramolecular crosslinks enough time to dissociate and form new connections. Crosslink dissociation and reformation is a viscoelastic process, increasing the tan δ. As the frequency increases, the crosslinks do not have enough time to dissociate (depending on the dissociation constant, k_d). The crosslinks start to act more fixed and the tan δ decreases. Conversely, covalent crosslinks are fixed at all frequencies. There are no crosslink dissociation constants to dictate the elasticity, so the networks are generally more elastic (tan δ closer to zero) and frequency independent.

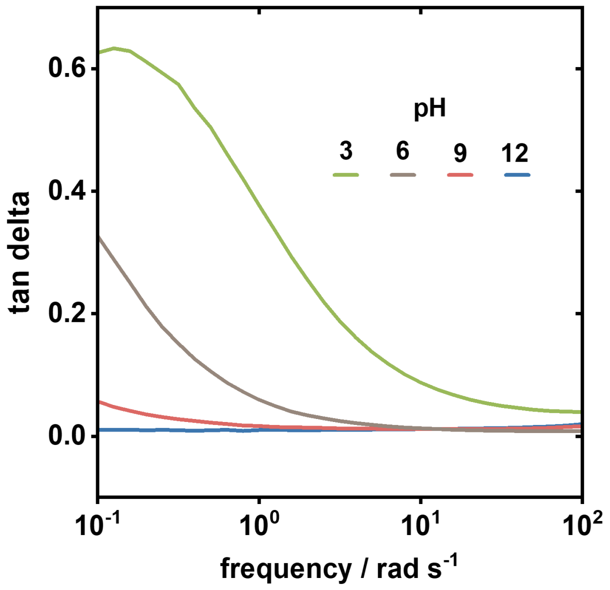

Figure S32 : Rheological frequency sweep measurements of tan δ at varying pH.

Under acidic conditions, the SPN displayed a frequency-dependent response with a high tan δ of > 0.6 at low frequencies, typical for a supramolecular hydrogel, Figure S32 . As the environmental pH is increased above the pK_a of the complex, the tan δ begins to drop and become more frequency independent. At a highly basic pH of 12, the SPN displays a completely frequency-independent response, maintaining a low tan δ throughout the measurement. Such observations indicate that at higher pH the polymer network is shifting from dynamic to static on account of the kinetic-locking of the crosslinks.

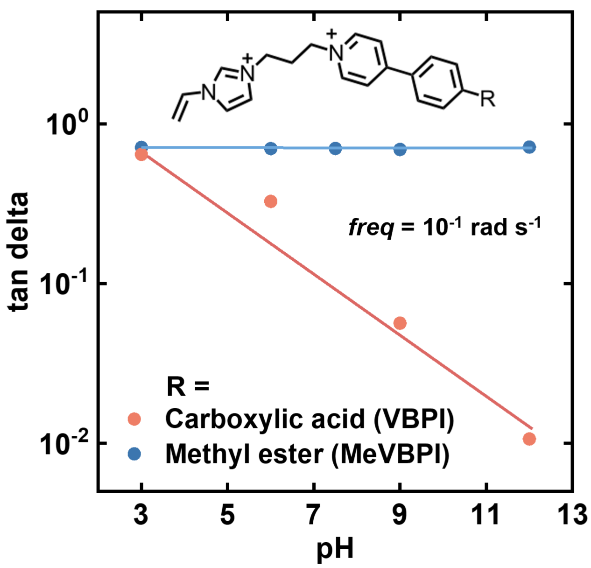

Figure S33 : Viscoelasticity for the MeVBPI and VBPI polymer networks versus pH at a frequency of 0.1 rad s^{-1}. Bromide counterions have been omitted for clarity.

To ensure that the pH responsive behaviour derives from the supramolecular crosslinks as opposed to the polymer backbone, a control experiment was performed in which the methyl ester guest analogue (MeVBPI) was integrated with the SPN instead of VBPI. Methyl ester does not readily undergo protonation or deprotonation within physiologically relevant pH ranges, and therefore no locking mechanism should occur. Tan δ measurements were performed at a frequency of 0.1 rad s^{-1} at various pH, Figure S33 . While SPNs incorporating the guest VBPI showed highly pH dependent tan δ behaviour, the tan δ for the SPN using MeVBPI remained stable across each pH, maintaining a level consistent with viscoelastic supramolecular materials.

8 .2 Variable temperature rheology

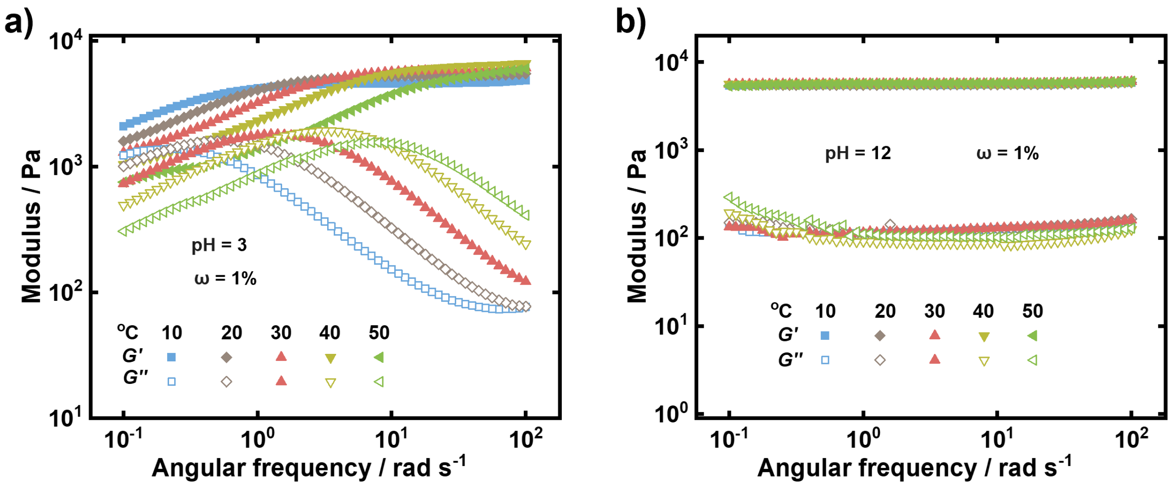

Figure S34 : a) Variable temperature rheology of the VBPI polymer networks ranging from 10 °C to 50 °C under acidic conditions (pH = 3). b) Variable temperature rheology of the VBPI polymer networks ranging from 10 °C to 50 °C under basic conditions (pH = 12).

Variable temperature rheology was performed to determine the effect of temperature on the polymer networks under locked and unlocked states. Under acidic conditions, the VBPI polymer networks rheological properties exhibit a considerable temperature dependence, whereby temperature has an effect on both the storage and loss modulus profiles. Such a temperature dependence is typical of dynamic polymer networks, whereby the kinetics of association and dissociation are affected by temperature.[S6] Meanwhile under basic conditions, the VBPI polymer networks show negligible temperature dependence, similar to that of polymer networks with fixed or covalent crosslinks . Such observations suggest that the polymer network is transitioning to a kinetically locked state under basic conditions.

Time-temperature superposition

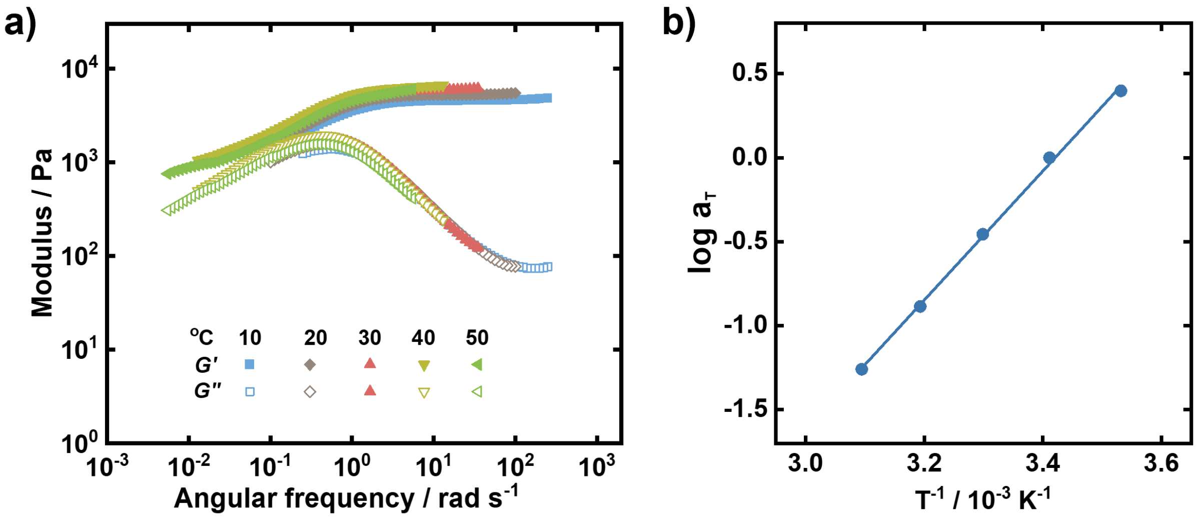

Figure S35 : Time-temperature superposition experiment (TTS) for the VBPI polymer networks under acidic conditions (pH = 3). a) TTS data in which frequency sweep measurements were conducted at a variety of temperatures. b) Plot of the linear shift parameter a_T against the inverse temperature (K^{-1}).

Energy dissipation within the VBPI polymer networks was investigated through time temperature superposition (TTS) experiments to obtain the activation energy (E_a) for local chain motion for the VBPI polymer networks under acidic conditions (pH = 3), Figure S35 . Fitting the temperature data to a horizontal shift parameter (a_T) resulted in an E_a of 17.6 kcal mol^{-1}, comparable to the unfolding barrier of the I27 domain of the human muscle protein *titin* (E_a = 17.0 kcal mol^{-1}).[S7] The shift factors can be correlated with the temperature using the Arrhenius equation as follows:

$$log(a_{Tref}) = \frac{E_a}{2.303R}\left(\frac{1}{T} - \frac{1}{T_{ref}}\right)$$

where E_a is the activation energy in kJ mol^{-1} and R is the gas constant (8.314 J K^{-1} mol^{-1}). The shift factors can be fitted linearly to the inverse of temperature (1/T). The activation energy is a measure of the energy barrier that must be overcome to allow for the relaxation of the viscoelastic VBPI polymer network.

8 .3 Stability testing

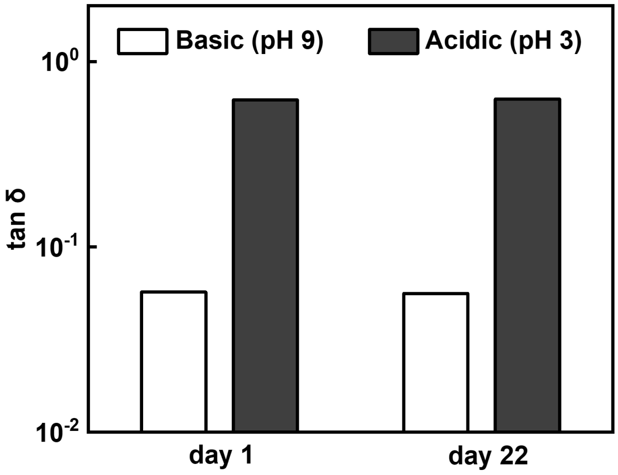

Figure S36 : Viscoelasticity tan δ values for the VBPI polymer networks after 22 days in acidic (pH 3) and basic (pH 9) environments.

The stability of the VBPI polymer networks in acidic (pH 3) and basic (pH 9) environments was investigated over a period of 22 days. As shown in figure S37 , negligible change is observed in the tan δ values of the polymer networks, suggesting that the viscoelasticity of the materials is unaffected. Such findings attest to the stability of the polymer networks against degradation.

8 .4 Stress relaxation

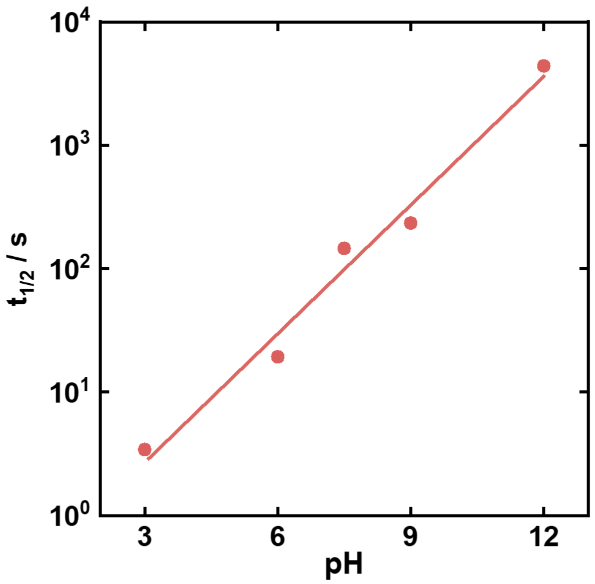

Figure S37 : Rheological stress relaxation half-time ($t_{1/2}$) for the VBPI polymer networks over a range of pH values.

Figure S37 shows the stress relaxation response of the bulk polymer network under stress for intermediary pH values. Stress relaxation measurements are related to the average lifetime of the crosslinks under stress. We found that the log of the relaxation half-time ($t_{1/2}$) of the network as a function of pH follows a linear relationship.

8 .5 Mechanical properties with pH

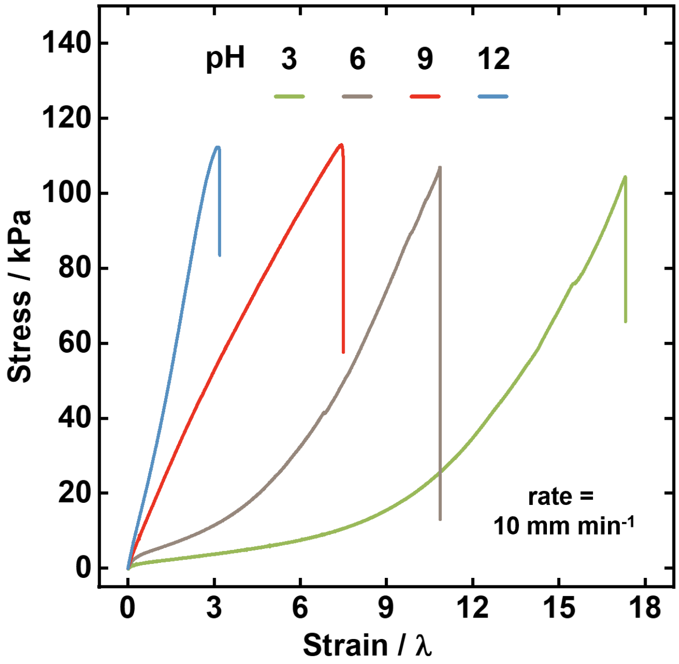

Figure S38 : Mechanical tensile testing of the polymer networks, showing stress-strain tensile curves at varying pH until network fracture.

The stretchability of the materials greatly diminishes with increasing pH, Figure S38 . This is consistent with a transition from a dynamic to static polymer network. Typically, dynamic networks display higher stretchability due to the energy dissipation and bond reformation of the crosslinks under strain, reducing the covalent bond scission that occurs with fracture. For non-dynamic materials, the crosslinks cannot dissociate and reform, resulting in highly concentrated stress points and scission of covalent bonds leading to fracture.[S8]

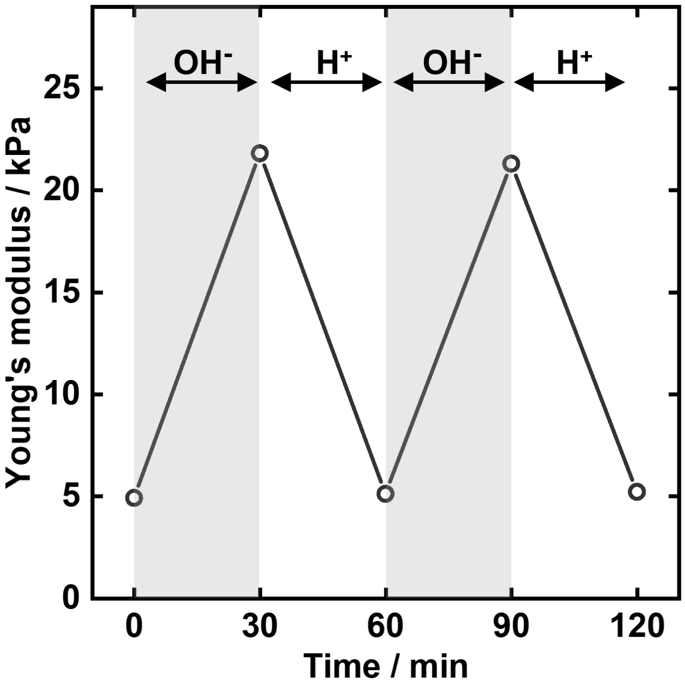

Figure S39 : Young's modulus of the VBPI polymer network following immersion in acidic (pH = 3) and basic (pH = 9) solutions for 30 minutes over multiple cycles.

Figure S39 shows the reversibility of the mechanical properties during cycling between acidic (pH = 3) and basic (pH = 9) conditions, whereby tensile tests up to 50% strain were performed on the materials following immersion in each pH environment after 30 minutes on a VBPI polymer network specimen. The materials demonstrated exceptional reversibility over five cycles, maintaining a stable Young's modulus consistent with that of the locked and unlocked crosslinking states.

9 pH-dependent Cargo Release Study

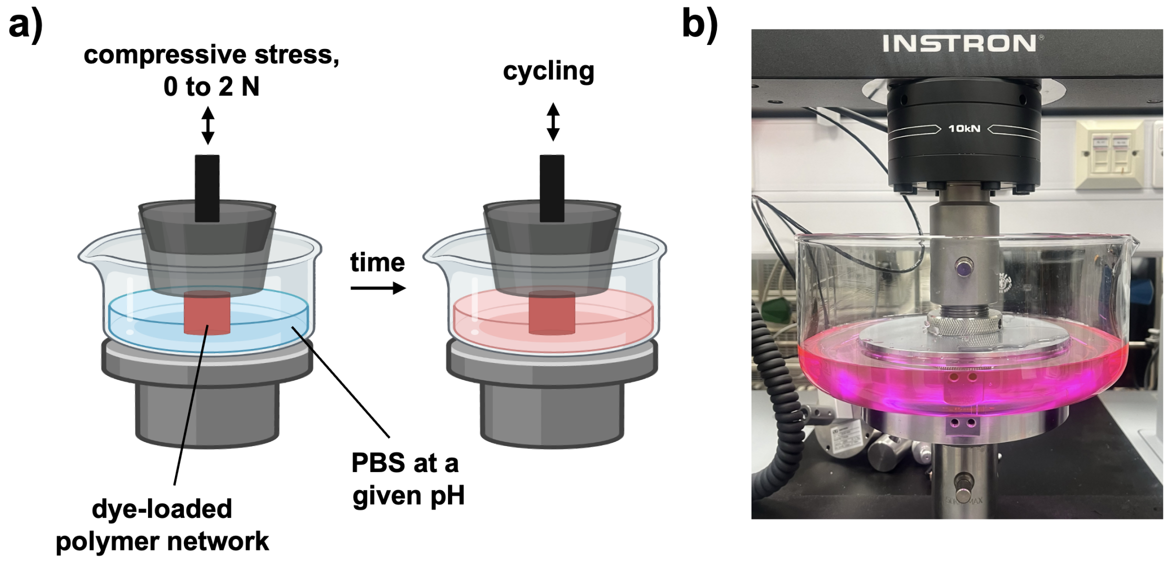

Figure S40 : a) Schematic of the experimental setup for the pH-dependent cargo release study. b) Photograph of the experimental setup for the pH-dependent cargo release study.

The VBPI polymer networks were prepared as described in section 7 . Following polymer network fabrication, the polymer networks were immersed in a dye solution containing 5 mg/ml Sulforhodamine B in phosphate buffer solution (PBS) for 6 hours, ensuring full diffusion of dye into the polymer network. Cyclic compressive experiments were then performed on an Instron machine (34TM-10) equipped with a 5-kN load cell, by immersing the polymer network in a solution of 500 ml PBS adjusted to the desired pH using either HCl or NaOH. The compressive force was cycled between 0 and 2 N at 1 mm/min over three hours. 0.2 ml aliquots of solution were taken every 30 minutes and tested for absorbance, and the concentration was calculated through a linear relationship between absorbance and concentration ($A = 65.73C + 0.0365$, where A is absorbance and C is concentration in mM), in order to quantify the release of the dye into the surrounding PBS solution.

References

(S1) Kim, J. New cucurbituril homologues: Syntheses, isolation, characterization and X-ray crystal structures of cucurbit[n]uril (n = 5, 7, and 8). *J. Am. Chem. Soc.* **2000**, *5*, 540–541.

(S2) Day, A.; Arnold, A. P.; Blanch, R. J.; Snushall, B. Controlling factors in the synthesis of cucurbituril and its homologues. *J. Org. Chem.* **2001**, *66*, 8094–8100.

(S3) Fulmer, G. R.; Miller, A. J.; Sherden, N. H.; Gottlieb, H. E.; Nudelman, A.; Stoltz, B. M.; Bercaw, J. E.; Goldberg, K. I. NMR chemical shifts of trace impurities: common laboratory solvents, organics, and gases in deuterated solvents relevant to the organometallic chemist. *Organometallics* **2010**, *29*, 2176–2179.

(S4) Leira-Iglesias, J.; Sorrenti, A.; Sato, A.; Dunne, P. A.; Hermans, T. M. Supramolecular pathway selection of perylenediimides mediated by chemical fuels. *Chem. Comm.* **2016**, *52*, 9009–9012.

(S5) Saleh, N.; Koner, A. L.; Nau, W. M. Activation and stabilization of drugs by supramolecular pKa Shifts: drug-delivery applications tailored for cucurbiturils. *Angew. Chem.* **2008**, *120*, 5478–5481.

(S6) Jourdain, A.; Asbai, R.; Anaya, O.; Chehimi, M. M.; Drockenmuller, E.; Montarnal, D. Rheological properties of covalent adaptable networks with 1, 2, 3-triazolium cross-links: the missing link between vitrimers and dissociative networks. *Macromolecules* **2020**, *53*, 1884–1900.

(S7) Liu, J.; Tan, C. S. Y.; Yu, Z.; Li, N.; Abell, C.; Scherman, O. A. Tough supramolecular polymer networks with extreme stretchability and fast room-temperature self-healing. *Adv. Mater.* **2017**, *29*, 1659.

(S8) Wang, S.; Hu, Y.; Kouznetsova, T. B.; Sapir, L.; Chen, D.; Herzog-Arbeitman, A.; Johnson, J. A.; Rubinstein, M.; Craig, S. L. Facile mechanochemical cycloreversion of polymer cross-linkers enhances tear resistance. *Science* **2023**, *380*, 1248–1252.
